# Optimised Machine Learning and Statistical Modelling for Predicting the Design Strengths of Hardened 3D Printed Concrete

**DOI:** 10.3390/ma19153358

**Published:** 2026-08-06

**Authors:** Mohamed N. Omar, Mustafa Batikha, Md Azher Uddin

**Affiliations:** 1 School of Energy, Geoscience, Infrastructure and Society, Heriot-Watt University, Dubai P.O. Box 501745, United Arab Emirates; mno2003@hw.ac.uk; 2 School of Mathematics and Computer Science, Heriot-Watt University, Dubai P.O. Box 501745, United Arab Emirates; m.uddin@hw.ac.uk

**Keywords:** 3D concrete printing, standardisation, statistical approach, machine-learning modelling, design strengths

## Abstract

3D Concrete Printing (3DCP) has emerged as a transformative construction technology, offering enhanced design flexibility, reduced material waste, improved construction efficiency, and safer working environments. Despite these advantages, the absence of standardised design provisions for structural 3DCP elements remains a major obstacle to its widespread adoption. This study develops statistical and machine learning models to predict the design strengths of hardened 3D-printed concrete. A comprehensive database of experimental results published between 2012 and 2025 was compiled and analysed, encompassing the compressive, flexural, and shear strengths of hardened 3DCP materials. Regression-based statistical modelling was employed to derive design strength equations and assess their consistency with conventional concrete design approaches. In parallel, several machine learning algorithms were developed using the same dataset to benchmark predictive performance and evaluate the robustness of the proposed statistical models. The statistical models achieved good predictive accuracy for compressive and flexural strengths, with coefficients of determination (R^2^) of 0.83 and 0.67, respectively. By contrast, the shear strength model exhibited greater variability (R^2^ = 0.39), reflecting the limited availability and considerable scatter of published experimental data. Benchmarking against the optimised machine learning model demonstrated excellent agreement with the proposed statistical equations, yielding coefficients of determination of 0.93 and 0.94 for compressive and flexural strengths, respectively. The proposed modelling framework provides preliminary design strength predictions for hardened 3D-printed concrete, contributing to the development of robust structural design guidelines and facilitating the wider adoption of 3DCP in construction practice.

## 1. Introduction

The construction industry currently exhibits relatively limited digital integration and heightened vulnerability to incidents. As urban populations continue to expand, there is an escalating demand for affordable housing, public transportation, and infrastructure [1,2]. Consequently, incorporating advanced technologies such as robotic construction and Additive Manufacturing (AM) becomes crucial to enhance automation within construction.

In addition, the adoption of this technology can provide a solution for countries grappling with labour shortages in the construction sector [3]. Likewise, increased digitisation would make the construction industry more appealing to younger generations and ensure safer working environments at construction sites [4]. When integrated into traditional construction, AM technology can bring about positive transformations in terms of cost, technology, and societal impact [5]. Early research suggests that 3D Concrete Printing (3DCP) offers design freedom, mass personalisation, construction time reduction, and production cost reduction [6]. Moreover, the implementation of 3DCP technology has garnered considerable attention in the construction industry due to its potential to contribute to sustainable building practices [7]. A key aspect of sustainability in 3DCP is the reduction in material waste. Traditional construction methods often generate substantial material waste due to the need to cut and shape materials to conform to specific dimensional requirements [8]. In contrast, 3DCP enables precise and accurate placement of materials, minimising waste and optimising material usage [9]. By reducing the volume of material required and mitigating waste generation, 3DCP promotes resource efficiency and contributes to a more sustainable construction process. Batikha et al. [1] conducted a study to evaluate the efficiency of 3DCP compared to other construction techniques, specifically in-situ Reinforced Concrete (RC), Cold-Formed Sections (CFS), Hot-Rolled Sections (HRS), and Prefabricated Modular Construction (PMC). The study concluded that 3DCP produces 25% and 42% fewer CO_2_ emissions than the in-situ RC and PMC methods, respectively. Moreover, the same research found that 3DCP is 10% cheaper than the in-situ RC technique and 21% less costly than PMC. Another sustainable advantage of 3DCP lies in its potential to enhance energy efficiency in construction [10]. The technique allows for the integration of complex geometries and sophisticated internal parts, such as hollow sections or optimised truss-like elements, which can significantly reduce the overall weight of printed components [11]. This lightweight construction not only reduces the energy required for transportation and installation but also decreases the demand for structural support systems [12]. Additionally, 3DCP offers the opportunity to incorporate insulation materials directly into the printed elements, improving thermal performance and reducing the energy consumption associated with heating and cooling in buildings [13]. By enabling energy-efficient designs and reducing operational energy requirements, 3DCP contributes to sustainable building practices and supports the goal of achieving environmentally friendly construction solutions [14].

Since 2012, the most frequently studied topics in 3DCP have been the selection of new and alternative printing materials, mixture composition and design, development and modelling of printability indicators, and evaluation of fresh and hardened properties [15,16].

In conventional construction, a large volume of concrete is poured, and minor changes in the concrete’s physical/chemical properties or mix design usually do not affect the end product’s performance [17]. However, with 3DCP, thin layers of 10 to 50 mm are continuously extruded, which means that slight variations in the compound result in a change in rheology that can affect buildability and cause structural failure [18,19]. Addressing this requires extensive interdisciplinary research to examine materials, processes, print parameters, and quality assurance (fresh and hardened states) [8]. With conventional construction, there is plenty of time to perform manual inspections and fix problems [20]. However, with 3DCP, the total time for the build process can be as short as a day [21]. It is also important that the designed elements are printed without interruptions to improve adhesion between the layers and to minimise the risk of material hardening in the printer’s delivery system (i.e., the pump, hose, or extruder) [22,23]. The available concrete printers used for research and industrial development mainly have open-loop controls [24,25]. In the absence of a control loop system, good 3DCP quality can only be obtained if the printing parameters are well-defined and the system is very robust [26]. However, construction material quality can vary from batch to batch, and any printable mix depends on various interdependent factors such as build speed, print path, curing, temperature, surface moisture, etc. [27]. The printer operator must rely on their experience to manually determine the above factors and visually inspect the print quality [28,29]. The challenge becomes more complex in large-scale printing and on-site construction, where environmental conditions such as temperature, humidity, and wind can become dominant factors [27,30]. As 3DCP development progresses from laboratory to in-situ development, understanding the test patterns for fresh and hardened properties of 3DCP and coupled factors that affect them will help maintain the quality of the layered structures [31].

**Figure 1:**
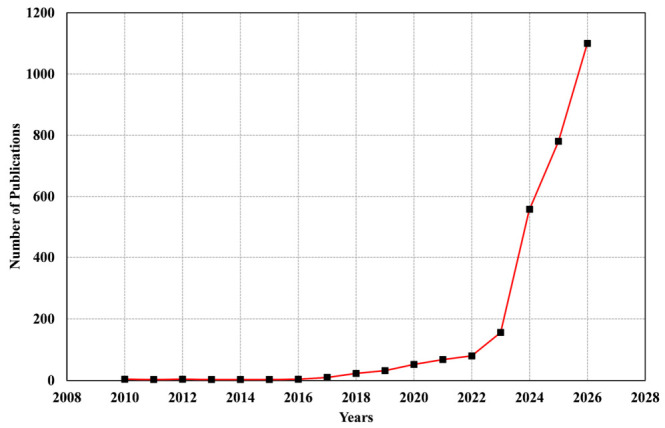
The growth of the 3DCP research.

Figure 1 illustrates the growth of 3DCP research from 2010 to 2026. While a gradual increase began after 2016, the field has recently seen a more pronounced expansion, with annual publications reaching 156 in 2023 and 558 in 2024 [32]. Projections suggest this output will exceed 1100 by the end of 2026 [33,34]. This recent acceleration significantly outweighs the total of 103 publications recorded between 2010 and 2019, reflecting 3DCP’s transition from an experimental interest to a dominant research domain in construction.

Carolo and Haines provided an early analysis of the 3DCP market through 2023, identifying 25 pioneering structures worldwide. Their findings established a clear shift in activity because 16 of those cases were initiated after 2019 [35]. However, the market has since moved beyond these early prototypes into a phase of industrial scaling. Evidence from 2024 and 2025 indicates a substantial departure from the individual pilot projects of the previous decade. The sector has recently transitioned to multi-unit residential developments and large-scale infrastructure. By early 2026, the number of completed 3D-printed buildings has grown to over five times the 2023 baseline [33,34]. This expansion reflects a maturing market that is increasingly capable of meeting global housing demands through automated construction.

Previous research on 3DCP has primarily focused on the printing process, testing techniques, and practical applications, with less emphasis on the design process and the standards used for designing 3DCP structures. As highlighted by recent comprehensive reviews [33,34], the industry currently lacks unified structural codes or design provisions, such as those established in the Eurocodes or ACI standards for conventional concrete, tailored specifically to the unique anisotropic mechanics of 3DCP. Furthermore, contemporary studies continue to rely heavily on isolated empirical testing of specific material mixes and interfaces, rather than developing generalised, safety-oriented design equations [36,37]. Consequently, engineers are often forced to adopt unverified assumptions or conduct costly, project-specific testing. This gap in 3DCP standardisation presents a critical challenge that needs to be addressed [38]. To address this research gap, this paper presents a novel objective. It introduces a unified data-driven predictive framework that integrates statistical modelling and machine learning approaches to establish preliminary strength provisions for 3DCP. Unlike previous studies that have predominantly focused on isolated experimental investigations or mix-specific formulations [36,37], this research develops and analyses an extensive global database of hardened 3DCP properties reported between 2012 and 2025. The proposed framework enables the prediction of compressive, flexural, and shear design strengths within a consistent analytical approach, representing one of the first attempts to derive preliminary design strength provisions for hardened 3D-printed concrete. Furthermore, the study identifies the current limitations of available experimental datasets, particularly regarding shear strength, and highlights critical research priorities required to advance the development of reliable structural design guidelines for 3DCP.

## 2. Research Methodology

**Figure 2:**
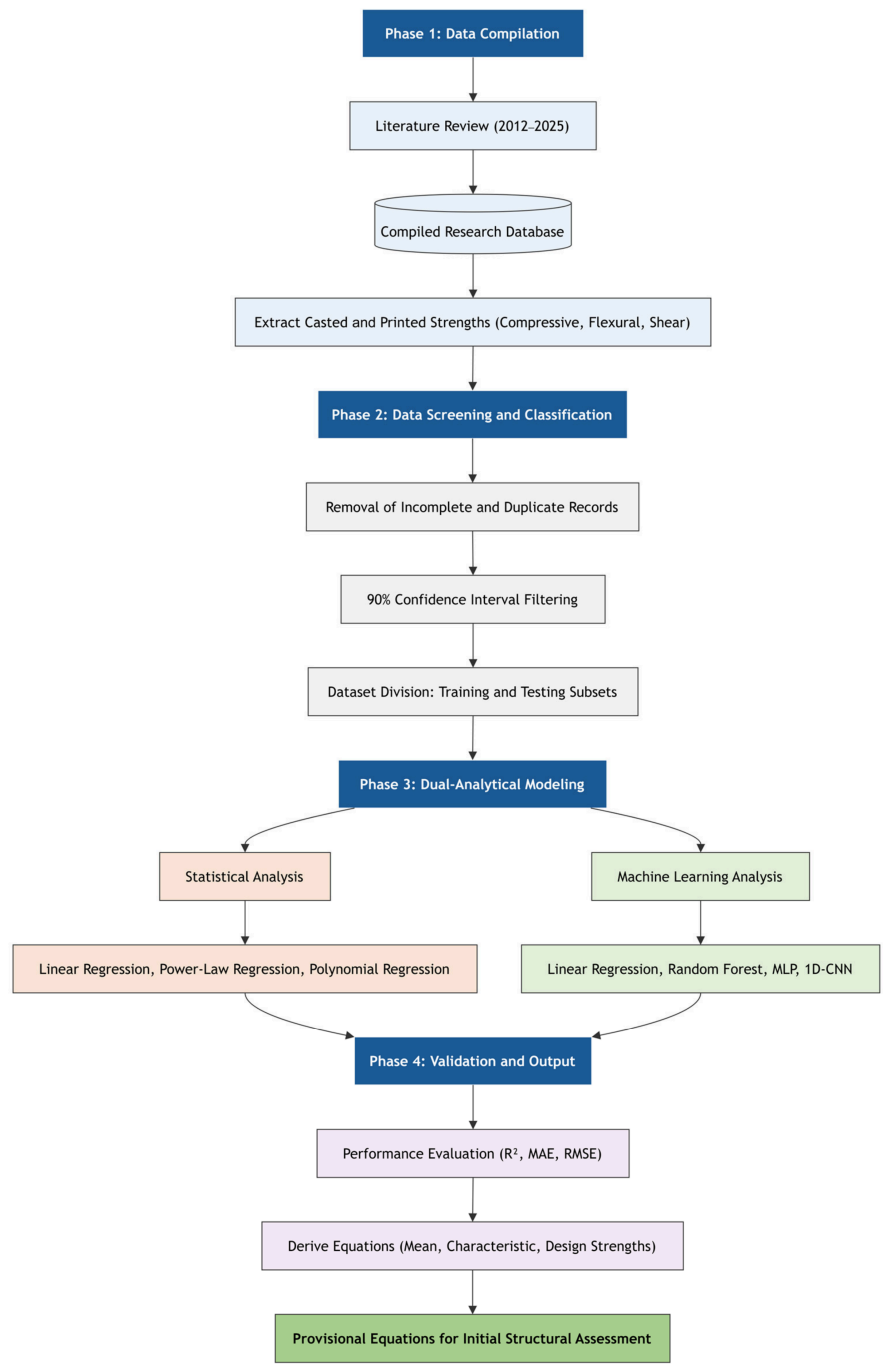
Research methodology framework for 3DCP strength prediction followed in this research.

This study adopts a data-driven framework to establish reliable predictive relationships between cast and printed mechanical properties of extrusion-based 3DCP. The research methodology consists of four main stages, including database compilation from the published literature, data screening and classification, statistical modelling to derive analytical relationships, and machine learning analysis for validation and comparative assessment. The overall approach aims to develop statistically robust and practically applicable equations that can support the structural implementation of 3DCP. To illustrate this progression from data compilation to the derivation of the provisional design equations, the research framework is visually summarised in Figure 2.

A comprehensive database (Table S1) [26,27,36,37,39,40,41,42,43,44,45,46,47,48,49,50,51,52,53,54,55,56,57,58,59,60,61] of experimental results related to extrusion-based 3DCP was compiled from peer-reviewed journal publications. The collected data include material composition, mix design parameters, chemical admixture dosages, printing and processing variables, and corresponding mechanical properties. Only studies reporting both printed and cast strength values were considered to enable direct correlation between conventional and printed performance. The compiled information covers material constituents such as cement content, supplementary cementitious materials (including fly ash, silica fume, ground granulated blast furnace slag, limestone powder, and metakaolin), fine aggregate content, and maximum aggregate size. Mix design parameters such as water content, water-to-binder ratio, sand-to-binder ratio, and total binder content were also extracted. In addition, admixture dosages including superplasticiser, viscosity-modifying agents, retarders, accelerators, and other additives were collected where available. To account for process-induced effects, printing parameters such as nozzle diameter, layer height, printing speed, time interval between layers, curing conditions, and ambient temperature were included. The incorporation of both material and process variables is essential, as the mechanical behaviour of 3DCP is influenced not only by mixture composition but also by interlayer bonding and printing-induced anisotropy [38,62]. However, it is worth noting that the aforementioned parameters, although collected in detail, were not utilised in this study, as the objective of the research was to develop safe and reliable preliminary design equations irrespective of the process employed to manufacture the final product. Nevertheless, the data gathered in this study will prove valuable for future investigations into the influence of specific parameters on printing quality.

To ensure the reliability and consistency of the database, the collected data were subjected to a systematic screening procedure. Duplicate records and incomplete entries lacking essential mechanical or compositional information were removed. Only results obtained using extrusion-based printing techniques were retained, while data from other additive manufacturing methods were excluded to maintain methodological consistency. Where multiple curing ages were reported, 28-day strength values were prioritised to ensure uniform comparison and to minimise age-related variability. Reported strength values were also examined for physical plausibility, and extreme outliers resulting from experimental inconsistencies or non-standard testing conditions were excluded. The final dataset, therefore, represents the most reliable and statistically consistent subset of available experimental results.

Following the screening process, the data were classified according to the type of mechanical response. Separate datasets were established for printed compressive strength, printed flexural strength, and printed shear strength. In addition, the corresponding cast compressive strength was used as the primary independent variable in the statistical analysis. Cast compressive strength was selected because it is widely used in engineering practice as a quality control parameter and provides a practical basis for estimating the structural performance of printed elements. To further investigate potential variations associated with technological development, the collected data were also categorised into different time periods, allowing the assessment of possible trends related to material optimisation and advancements in printing technology.

Statistical analysis was performed to establish empirical relationships between cast compressive strength and the corresponding printed mechanical properties. To achieve this, the data were analysed using a normal distribution framework [63,64]. A 90% confidence interval (z-score = 1.645) [65,66,67,68] was employed to identify and exclude anomalous data points and to establish structural safety limits, consistent with established practices in engineering data analysis. The coefficient of determination (R^2^) was subsequently used to assess the goodness-of-fit between the observed data and the fitted regression model [69,70,71,72]. Power-law regression models were adopted to capture the nonlinear relationship between these parameters, which is consistent with the behaviour commonly observed in cementitious materials. For each mechanical property, the regression coefficients were determined by minimising the prediction error between experimental and calculated values. Model performance was evaluated using the coefficient of determination (R^2^) and associated error indicators. The resulting relationships provide simplified analytical expressions that can be readily used for preliminary design and structural assessment.

To further evaluate the predictive capability of the derived statistical relationships, supervised machine learning models were developed using the filtered database. Machine learning techniques are capable of capturing complex nonlinear interactions among multiple variables and have been successfully applied in predicting the mechanical properties of concrete [73,74]. Prior to model development, all numerical features were normalised to improve computational efficiency and prevent bias due to differences in variable magnitude [75]. The dataset was randomly divided into training and testing subsets to assess the generalisation performance of the models. Three algorithms were implemented, including Random Forest, Multilayer Perceptron, and a one-dimensional Convolutional Neural Network. Model performance was evaluated using the coefficient of determination (R^2^), mean absolute error (MAE), and root mean square error (RMSE). Due to the limited availability of reliable shear strength data in the literature, machine learning analysis was performed only for compressive and flexural strength prediction, while shear behaviour was evaluated using the statistical modelling approach. The predictions obtained from the machine learning models were subsequently compared with the analytical equations to assess consistency, robustness, and practical applicability across the structural strength range considered in this study.

### Database Compilation and Screening Strategy

To construct a comprehensive database of extrusion-based 3D Concrete Printing (3DCP), a systematic literature search was conducted covering publications from 2012 to 2025. The search encompassed the major scientific indexing databases of Scopus, Web of Science, ScienceDirect, and Google Scholar. Boolean search operators were employed to combine the primary and secondary keywords: (“3D concrete printing” OR “3DCP” OR “extrusion-based additive manufacturing”) AND (“compressive strength” OR “flexural strength” OR “shear strength” OR “interlayer bond strength”) AND (“casted” OR “moulded” OR “conventionally cast”).

**Figure 3:**
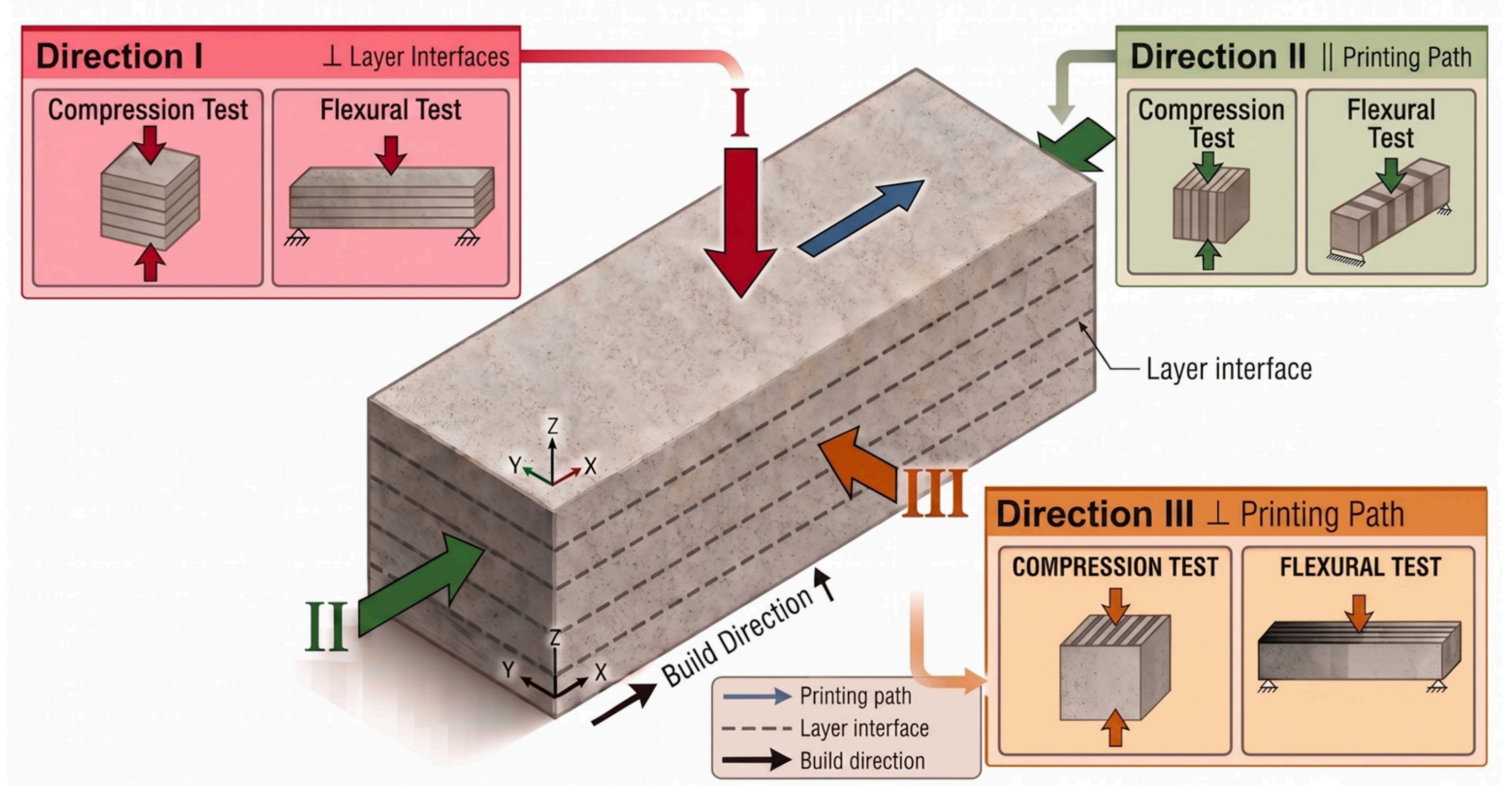
Testing directions of 3DCP specimens for compression and flexural testing.

- **Inclusion criteria** were defined as follows: (i) peer-reviewed journal articles and conference proceedings published between 2012 and 2025; (ii) experimental investigations involving extrusion-based 3DCP mixtures; (iii) studies reporting paired experimental results for conventionally cast control specimens and 3D-printed specimens produced from the same mixture formulation and tested at the same curing age; and (iv) clear identification of specimen loading orientations (e.g., perpendicular, lateral, and longitudinal) (see Figure 3).
- **Exclusion criteria** comprised: (i) non-extrusion-based additive manufacturing techniques (e.g., powder-bed binder jetting); (ii) numerical or analytical studies without corresponding experimental data; (iii) studies lacking conventionally cast reference specimens; and (iv) duplicate datasets reported in multiple publications.

Following the systematic search and screening process, 29 publications satisfied the selection criteria and were retained for database development (see Table S1). From these studies, a total of 225 paired experimental observations were extracted, comprising 116 compressive strength pairs, 86 flexural strength pairs, and 23 shear strength pairs.

To minimise the influence of extreme observations that may arise from experimental execution errors, specimen defects, or inconsistencies in testing procedures reported across different studies, a statistical outlier screening procedure was performed. For each mechanical property (compressive, flexural, and shear), the ratio of printed-to-cast strength was calculated. Assuming an approximately normal distribution of these ratios, a central 90% reference interval was established using the following equation:

(1) $${CI}_{90\%}=\mu \;\pm 1.645\;\sigma$$

where μ represents the sample mean of the strength ratio and σ denotes the corresponding sample standard deviation. Observations falling outside these statistical limits were classified as extreme values and excluded from the subsequent modelling. The 90% reference interval was selected to reduce the influence of anomalous observations while preserving sufficient experimental data for robust statistical analysis.

Following the filtering procedure, the database was reduced to a final dataset of 201 paired experimental observations, comprising 101 compressive strength pairs, 78 flexural strength pairs, and 22 shear strength pairs. This filtered dataset was subsequently used for the development and evaluation of the proposed statistical and machine learning models.

## 3. Mechanical Properties of 3DCP Specimens

As of now, the understanding of the mechanical properties of 3DCP remains limited [59]. However, notable research on extruded concrete materials suggests a reduction in porosity, leading to enhanced strength and stiffness [76]. This section aims to explore the documented mechanical properties of 3DCP, providing insights into its distinct characteristics and performance.

### 3.1. Compressive and Flexural Strength

While the water–cement (w/c) ratio plays a crucial role in determining the concrete paste strength, it is important to note that air entrainment can significantly diminish it, as supported by extensive research [77,78]. Additionally, factors such as curing methods and the direction of testing in relation to interlayer joints also wield influence on the overall strength [59,79,80]. These variables demand careful consideration when interpreting the results of compressive and flexural tests conducted on 3DCP specimens. Moreover, control specimens prepared through standard casting and curing procedures are included for comparison, as summarised in Table 1 by various researchers [59,81].

In a study by Nerella and Mechtcherine [82], increased compressive and flexural strengths were observed in 3DCP specimens compared to cast specimens (Table 1). The samples were extracted by sawing and coring from 3DCP elements in different orientations, as depicted in Figure 3. However, in another study by Le et al. [61], lower compressive strengths were observed for specimens collected from 3DCP samples compared to cast specimens, as shown in Table 1. These contradicting results could be attributed to various mechanisms, including reduced air content and segregation due to non-optimised material printing and curing conditions. It is plausible that the reduced air content could have contributed to the strengthening of the specimens [82]. Le et al. [61] stated that moist-cured printed specimens were placed under damp hessian, while the cast specimens were cured at 20 °C after stripping at the age of one day until the test age of 28 days, which could explain the lower strength development in printed specimens. However, it is also plausible that defects in the specimens due to segregation may have caused the lower strength in printed specimens [83,84,85].

The orientation of the loading direction in a printed specimen significantly impacts its strength properties, as demonstrated in Table 1 [59,84,86,87,88,89]. According to Feng et al. [87], when tested parallel to the layer deposition [direction I in Figure 3], compressive strength was higher than when tested perpendicular to the layer deposition [59]. Though this phenomenon is challenging to explain, it is noteworthy that Feng et al. [87] heat-cured their specimens and conducted the tests after only three hours. Feng et al. [87] also investigated the failure mechanism of printed cubes under compression testing. In all cases, the specimens displayed diagonal failure, with two sets of triangular cracks intersecting near the centre, forming an hourglass shape on the two opposite sides. The results of Nerella and Mechtcherine [82] and Feng et al. [87] provide clearer insights into the influence of joint position. Both studies consistently showed that compressive and flexural strengths were at their lowest when tested in direction III (Figure 3), corresponding to loading parallel to the joints. In this orientation, the 3DCP specimens experienced compressive and flexural splitting along the weaker joints under compressive and flexural loading, respectively.

**Figure 4:**
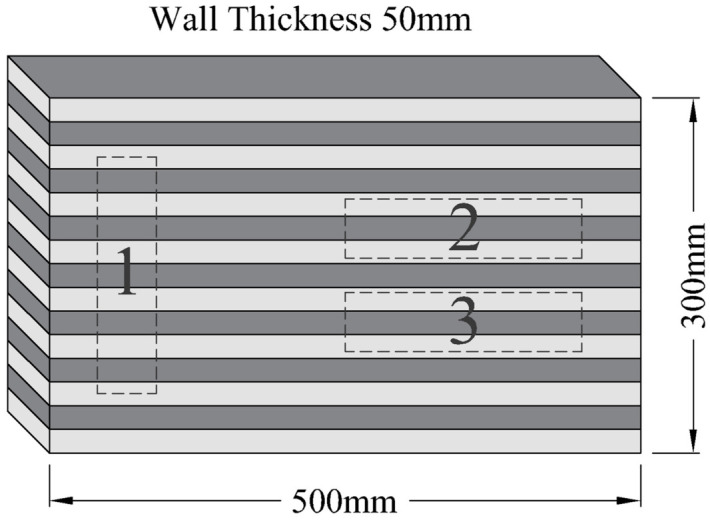
Extraction of specimens for mechanical testing from different orientations within a printed wall: (1) vertical orientation, (2) horizontal orientation in the upper section (altered printing quality), and (3) horizontal orientation in the lower section (initial printing quality).

An illustrative example is depicted in Figure 4, featuring a 3DCP wall produced from a single batch of concrete. The wall measures 500 × 300 mm with a thickness of 50 mm [90]. Initially, the printing quality is deemed satisfactory; however, over time, the hydration process intensifies, causing the matrix to become more challenging and altering the initial and final setting times. Additionally, some moisture may escape from the mixture, leading to a deterioration in printing quality. Consequently, inferior-quality concrete layers and interfaces may have formed in the upper sections. This inconsistency in quality can account for the variation in mechanical properties observed in the specimens obtained from Samples 1, 2, and 3, as depicted in Figure 4, thereby explaining the reported scatter by Le et al. [61]. Based on the discussion, it is hypothesised that in 3DCP, the load-bearing capacity of printed objects is significantly influenced by the printing direction and duration. This highlights the importance of considering the anisotropic properties of 3D-printed objects and selecting appropriate printing speeds when designing such structures [89,91,92].

**Table 1:** Comparison of mechanical properties of cast and printed objects.

Authors	Printed Element	Type of Test	Specimen Size	Testing Direction (Figure 2)	Testing Time of Specimens				
					Printed			Casted	
					3 Days	21 Days	28 Days	21 Days	28 Days
Le et al. [61]	350 × 350 × 120 mm—printed slab	Compressive strength (MPa)	100 mm cut cubes	I	-	-	96	-	107
				II			93		
				III			93		
	500 × 350 × 120 mm—printed slab		100 mm cut cubes	I	-	-	102		
				II			102		
				III			91		
	Trial printed curvy bench		63 mm cut cubes	I	-	-	74		
				II			82		
				III			76		
	500 × 350 × 120 mm—printed slab	Flexural strength (MPa)	400 × 100 × 100 mm cut specimens	I	-	-	16.5	-	11
				II			13		
				III			6.5		
	Trial printed curvy bench		220 × 63 × 50 mm cut specimens	I	-	-	12		
				II			13		
Feng et al. [87]	Printed cubes with a layer thickness of 0.0875 mm.	Compressive strength (MPa)	70.7 mm printed cubes		Testing 3h after casting			-	-
				I	11.2				
				II	7.23				
			50 mm printed cubes	I	16.8				
				II	13.2				
				III	11.6				
Nerella and Mechtcherine [82]	1000 × 300 × 38 mm—printed wall	Compressive strength (MPa)	35mm cut cubes	I	45.9	83.5	-	73.4	-
				II	49.7	80.6			
		Flexural strength (MPa)	160 × 35 × 35 mm cut specimens	I	4.8	5.8	-	5.1	-
				III	4.3	5.9			

### 3.2. Shear Strength of 3DCP Specimens

The shear strength of 3DCP is a crucial mechanical property directly influencing the structural performance and reliability of printed components [93]. Shear strength refers to the material’s ability to resist forces parallel to the plane of applied stress [94]. In the context of 3DCP, several factors influence shear strength, including printing parameters, material properties, and printing techniques [18,95,96,97]. Few studies have investigated the shear strength of 3DCP using different printing configurations and materials. For instance, Alchaar and Al-Tamimi [53] addressed the challenges associated with evaluating the bond strength of 3DCP specimens using an axial tension machine, such as gripping issues, setup complexity, and force eccentricity. They proposed a novel, simplified setup utilising a compression testing machine with T-shaped and U-shaped in-house manufactured steel fixtures. The study involved printed specimens comprising three stacking layers tested in a 90° orientation, with control specimens cast to match the printed ones. The investigation considered varying printing time intervals (30 s, 2.5 h, and 4 h) to approximate cement’s initial and final setting times. Control specimens exhibited an average Bond Shear Strength (BSS) of 5.71 MPa, while printed specimens showed decreasing BSS with longer time intervals. The study emphasised the importance of moisture for bond development in 3DCP and revealed the influence of material hardening on bond shear strength reduction over time. Notably, the presence of fibres in moulded specimens contributed to BSS, while their impact in printed specimens was minimal. The research highlighted accelerated hydration reactions in hot mixes, leading to rapid material hardening and potential challenges in bond development under specific exposure conditions. Visual assessments of interfacial regions after failure provided insights into variability factors affecting bond strength, including void formation and surface roughness.

In another study, Meurer and Classen [51] extensively investigated the shear strength of drill cores extracted from 3DCP specimens, addressing challenges in existing shear test setups. The study notes a significant decrease in shear strength for printed specimens in the interface region (approx. 1.8 N/mm^2^ for a 1-min interval time) compared to casted reference tests (4.6 N/mm^2^). The study investigates the effect of interval time (1 min to 40 min) but encounters reliability issues due to concrete mixing problems, with increased moisture content and enhanced shear strength at specific intervals. The authors advocate for further investigations to understand the influence of fresh concrete and interval time on the shear fracture process, expecting a decrease in shear strength with increased interval time.

In their study on the shear strength of 3DCP specimens incorporating ballast fibres in the concrete mix, Ma et al. [58] employed a double shearing test with a loading rate of 50 N/s until failure. The results indicated that the tested printed specimens in the direction parallel to the printing direction (III in Figure 2) exhibited about 10% less shear strength than the mould-cast samples. In contrast, the tested printed samples in directions perpendicular to the printing direction experienced shear strengths approximately 23% and 63% higher than the casted mortar. The highest average value was in direction I of Figure 2. It is worth noting that the shear strength of the casted mortar was 5.21MPa. The study suggested that loading in directions perpendicular to the printing direction transferred the shearing force across cold joints, minimising the impact of weak interfaces on overall mechanical properties. The research emphasised the enhancement in the shear capacity when fibres were aligned perpendicular to the shearing front, especially in the direction of gravity. This enhancement was attributed to the compactness of microstructures under the self-weight of the materials.

Rahul et al. [57] investigated the shear strength of 3DCP specimens, employing 10% silica fume of the binder content, 0.3% attapulgite clay of the binder, and 0.1% hydroxypropyl methylcellulose of the binder as special additives in three different mixes. The test revealed that the bond shear strength was lower by 29.2%, 26.4%, and 22.5% for silica fume, attapulgite clay, and hydroxypropyl methylcellulose, respectively. These findings align with observed trends in porosity measurements. Notably, the study emphasised the dependence of bond strength on print parameters, particularly the time gap between layer depositions (two minutes for horizontal layers and eight minutes for vertical layers). It was also acknowledged that preventing drying could mitigate strength reduction at the interface; however, the study did not implement specific measures to control drying. All experiments were conducted under ambient conditions of 30 °C and 70% relative humidity.

In another study, Razzaghian et al. [98] investigated the effect of incorporating nano-silica particles in the printing material on the shear strength of 3D-printed concrete panels. The research findings revealed that incorporating nano-silica particles improved the interfacial bonding between printed layers, resulting in enhanced shear strength and crack resistance. Nano-silica (NS) stands out as a frequent nano-additive used by researchers to modify normal cementitious composites, owing to its valuable physical and chemical properties. Due to its nano-scale size, nano-silica effectively fills the pores in the mixture matrix, generating additional C-S-H gel through its nucleation effect. This leads to a denser microstructure, reduced permeability, and heightened mechanical strengths, particularly compressive strength and inter-layer bond. Importantly, nano-silica plays a vital role in hindering the propagation of microcracks in the matrix, consequently increasing the capacity and ductility of the cementitious composite. This demonstrates the potential of using nanomaterials to enhance the mechanical properties of 3DCP structures [7,99].

Ahmed et al. [100] conducted an in-depth investigation into the shear and bond strength between layers in 3DCP structures, characterised by side-by-side, spaced, and cumulative successive filaments. Inherent interfaces in 3DCP, unavoidable due to the printing process, represent weak regions impacting the structural performance and long-term durability of 3D printed (3DP) elements. The adhesion strength of these interfaces, and consequently the mechanical properties of 3DP structures, is influenced by numerous material and processing parameters, including interlayer interval time, layer orientation, distance between the printing nozzle and working surface, rheology, thixotropy of the mix, and surface moisture. Additional factors such as the inertia of the extruded filament, printer limitations, filament distortion, and other robotic constraints further contribute to the complexity. The interlayer time interval, correlating the printing process, geometrical parameters, and material properties, necessitates optimisation to strike a balance between ensuring sufficient green strength for load-bearing capacity and promoting moist surfaces for robust interlayer adhesion. Chu et al. [101] recommended an optimum time gap of 10 min, emphasising its critical role in preventing weak structures and cold joint formation. Understanding the influence of material processing on extrusion cycle time requires meticulous optimisation iterations and co-simulation during design to ensure the desired structural performance is achieved. While previous studies have addressed bond behaviour in 3DCP, the challenge of filament-to-filament interfaces and cold joint formation remains a research gap. Research efforts have predominantly focused on enhancing bond strength at layer-to-layer interfaces, emphasising the need to explore filament-to-filament interfaces further. The study reveals that the interlayer bond in 3DCP is significantly affected by structural build-up velocity, with delay time (0 and 120 min) exhibiting minimal impact on mechanical properties under certain conditions but displaying a negative effect when parallel layer interfaces and increased delay times are present. Overall, the problematic interactions and challenges associated with interlayer and filament-to-filament bonding in 3DCP structures underscore the ongoing need for comprehensive research to enhance the performance and durability of 3DP constructions.

Overall, the shear strength of 3DCP is a complex property examined by a few studies and requires further research.

## 4. Results and Discussion

Following the research methodology outlined in Section 2, the data collected in Table S1 were treated as a comprehensive dataset spanning from 2012 to 2025. This unified approach allows for a holistic examination of the relationship between stress types and strength values, capturing the full technological evolution of 3DCP. To achieve the highest level of predictive accuracy, the data underwent a dual-stage analytical process involving both optimised statistical regression and Machine Learning (ML) analysis.

In the subsequent stage of analysis, a refined approach was adopted, involving a one-time filtering process to exclude any data points that exceeded the predetermined upper and lower bounds [102,103]. This filtering step aimed to eliminate outliers or extreme values that could potentially introduce bias or distort both the statistical and ML models. By removing such outliers, the resulting dataset became more focused and representative, allowing for a more accurate and robust examination [104]. For example, Table 2 identifies certain abnormal data points where the printed strength is recorded as being significantly higher than the casted concrete strength; the filtering process ensures these values are excluded to maintain the integrity of the predictive models.

The design equations representing the relationship between casted and printed strengths were derived through statistical optimisation to identify the mathematical formulations that yielded the highest value of (R^2^), particularly for flexural and shear strengths. Specifically, the mean and lower bound values from the filtered data were utilised to establish characteristic equations that prioritise structural safety. These optimised results were then compared with the ML analysis to determine the most reliable design values.

It is worth mentioning that this analytical framework was applied to all the collected data at once, regardless of the influence of mix design, concrete type, curing conditions, or printing directions. This would substantially reduce the number of observations available within each subgroup, particularly for flexural and shear strengths, thereby limiting statistical robustness and generalisability. Instead, the proposed framework intentionally incorporates the variability reported in the international literature to derive conservative characteristic design strengths applicable across the broad range of current 3DCP technologies. This philosophy is consistent with the objective of structural design codes, which prioritise safety and general applicability over optimisation for specific manufacturing conditions.

### 4.1. Compressive Strength

This section aims to present a statistical analysis of the compressive strength data. In deriving strength equations, the selection criteria involve referencing the current standard codes. For the case of compressive strength, the reference code employed is Eurocode 6 (EC6) [105]. The construction of 3DCP structures shares similarities with unreinforced bearing masonry walls of block units and bonding layers, with the bonding layer being the mortar; however, in 3DCP structures, all layers and bonding material consist entirely of concrete. The formula for the characteristic compressive strength of masonry, as outlined in EC6 (3.6.1.2) Equation (3.2), is as follows:

(2) $$f_{k}=k\;f_{b}^{0.7}\;f_{m}^{0.3}$$

where $f_{k}$ is the characteristic compressive strength of the masonry in N/mm^2^, k is a constant according to Table 3.3 in EC6 (3.6.1.2), and it is equal to 0.55 considering general-purpose mortar, $f_{b}$ is the normalised mean compressive strength of the units in the direction of the applied action effect in N/mm^2^, and $f_{m}$ is the compressive strength of the mortar in N/mm^2^. In the case of 3DCP structures, the equation representing the characteristic compressive strength can be rewritten as Equation (3) since $f_{b}$ equals $f_{m}$ because both materials are made of the same concrete.

(3) $$f_{ck}=0.55\;f_{b}$$

where $f_{ck}$ is the characteristic compressive strength of the concrete in N/mm^2^, and $f_{b}$ is the compressive strength of the mortar in N/mm^2^. Likewise, the equation derived from the statistical analysis will adhere to the formula outlined in EC6. It can be rewritten as follows:

(4) $$f_{ckp}=R_{Lower}\;f_{b}$$

where $f_{ckp}$ is the characteristic compressive strength of the printed concrete in N/mm^2^, and $R_{Lower}$ is the lower bound value estimated from the statistical analysis of the collected data.

**Figure 5:**
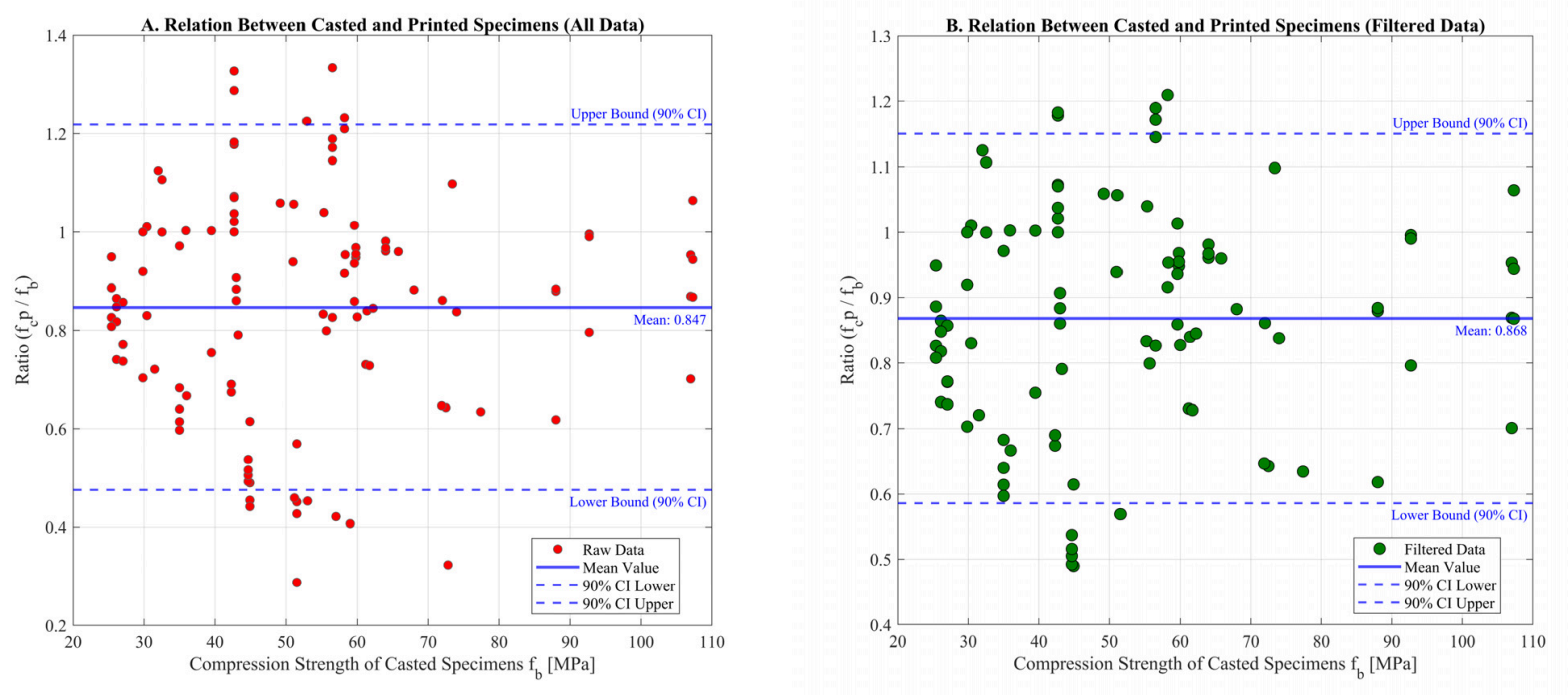
Statistical analysis of compressive strength data: (**A**) All collected data, (**B**) Filtered data.

**Figure 6:**
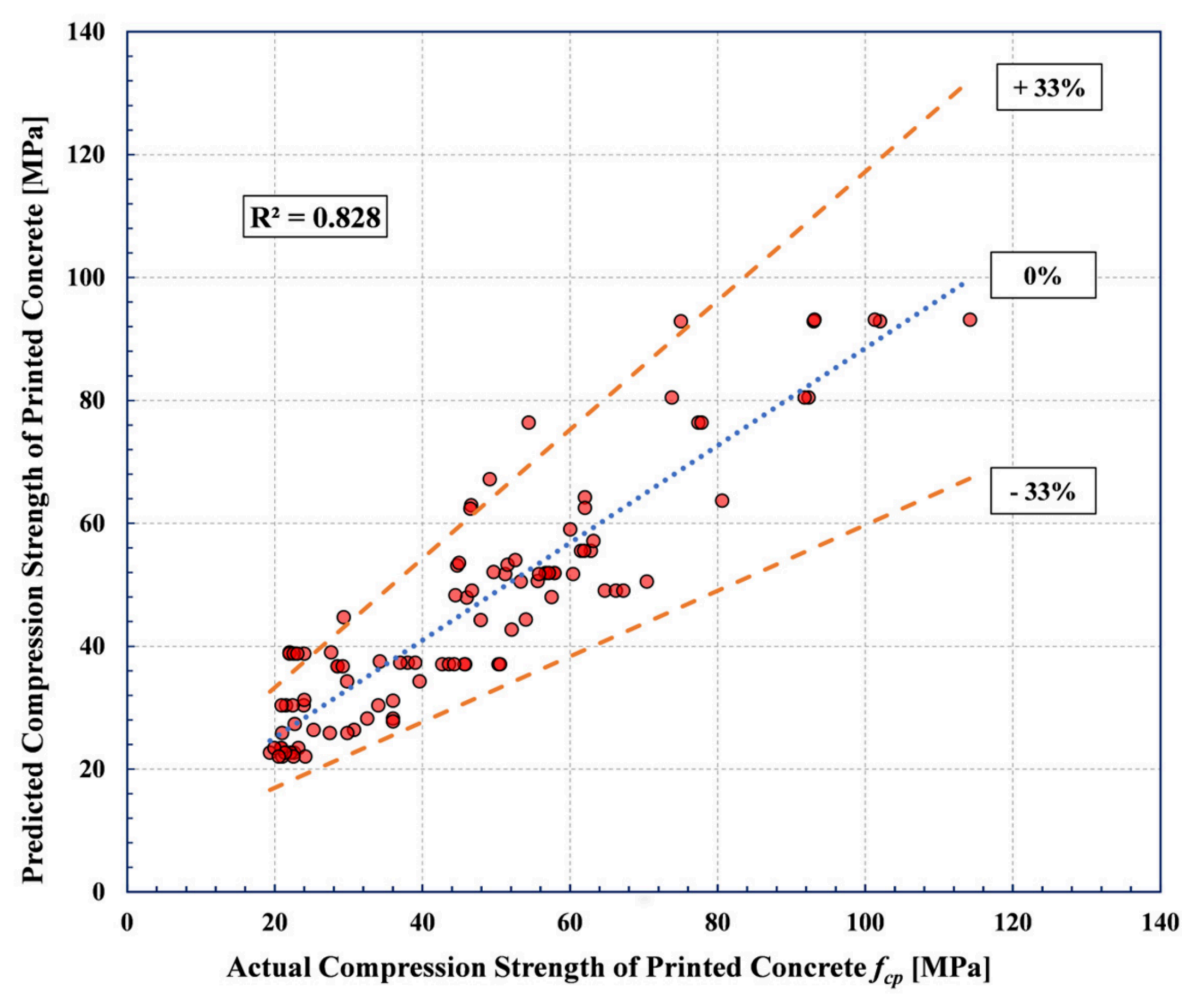
Relation between the actual compressive strength of the printed specimens and the predicted strength.

Figure 5 displays the outcomes of a statistical analysis conducted on the entirety of the collected data (Figure 5A) and a subsequent analysis following a one-time filtering process using a 90% confidence interval to remove anomalous data points as described in Section 4 (Figure 5B). This filtering approach is a recognised statistical technique for ensuring the reliability of experimental datasets by isolating significant results from random noise and identifying outliers that fall outside the expected probability distribution [106,107]. This analysis was performed using MATLAB (v2024b) software [108], known for its robust capabilities in data analysis and visualisation [109]. By mapping the outcomes of Figure 5 onto Equation (4), the predicted compressive strength of the printed samples can be estimated, adhering to established protocols for structural data validation and quality control as follows:

(5) $$f_{cp}=0.868\;f_{b}$$

(6) $$f_{ckp}=0.586\;f_{b}$$

where $f_{cp}$ is the mean compressive strength of the printed concrete elements in N/mm^2^, $f_{b}$ is the compressive strength of the casted samples and $f_{ck_{P}}$ is the characteristic compressive strength of the printed concrete in N/mm^2^. Figure 6 shows the relationship between the actual compressive strength demonstrated by the printed specimens and the strength values anticipated through Equation (5).

### 4.2. Flexural Strength

**Figure 7:**
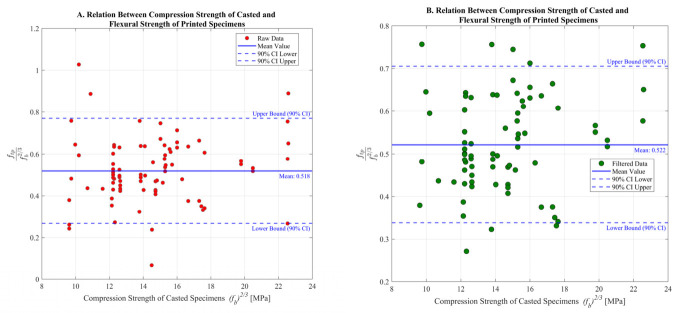
Statistical analysis of flexural strength data: (**A**) All collected data, (**B**) Filtered data.

In line with Section 4.1, a statistical analysis was conducted on the collected flexural strength data for both casted and printed specimens. The tensile strength in Table 3.1 of EC2 [110] is given as follows:

(7) fctm=0.3 fck23fctk=0.7 fctm=0.21 fck23 (5% fractile)

where $f_{ctm}$ is the mean flexural strength in N/mm^2^, $f_{ck}$ is the characteristic compressive strength in N/mm^2^, and $f_{ctk}$ is the characteristic flexural strength in N/mm^2^. Equation (19.2.3.1) in the ACI 318-19 [111] states the modulus of rupture as follows:

(8) fr=0.62 λ fc′ 12fctk=∅fr=0.434 fc′ 12 (5% fractile)

where $f_{r}$ is the modulus of rupture in N/mm^2^, λ is the aggregate factor, and it is equal to 1.0 in the case of normal-weight concrete, $f_{c}^{\prime }$ is the characteristic compressive strength in N/mm^2^, and ϕ is a safety factor equal to 0.7 based on Section 17.6.1.1 of the ACI 318-19 standard for calculating the characteristic flexural strength (5% fractile). The predicted flexural strength equation from the statistical analysis will conform to the formulation specified in EC2 and take the following shape:

(9) ftkp=RLower fb23

where $f_{tkp}$ is the characteristic flexural strength of the printed concrete in N/mm^2^, $R_{Lower}$ is the lower bound value estimated from the statistical analysis of the collected data, and $f_{b}$ is the compressive strength of the mortar in N/mm^2^. Figure 7 describes the statistical analysis of the complete dataset and the analysis after applying the appropriate filter.

**Figure 8:**
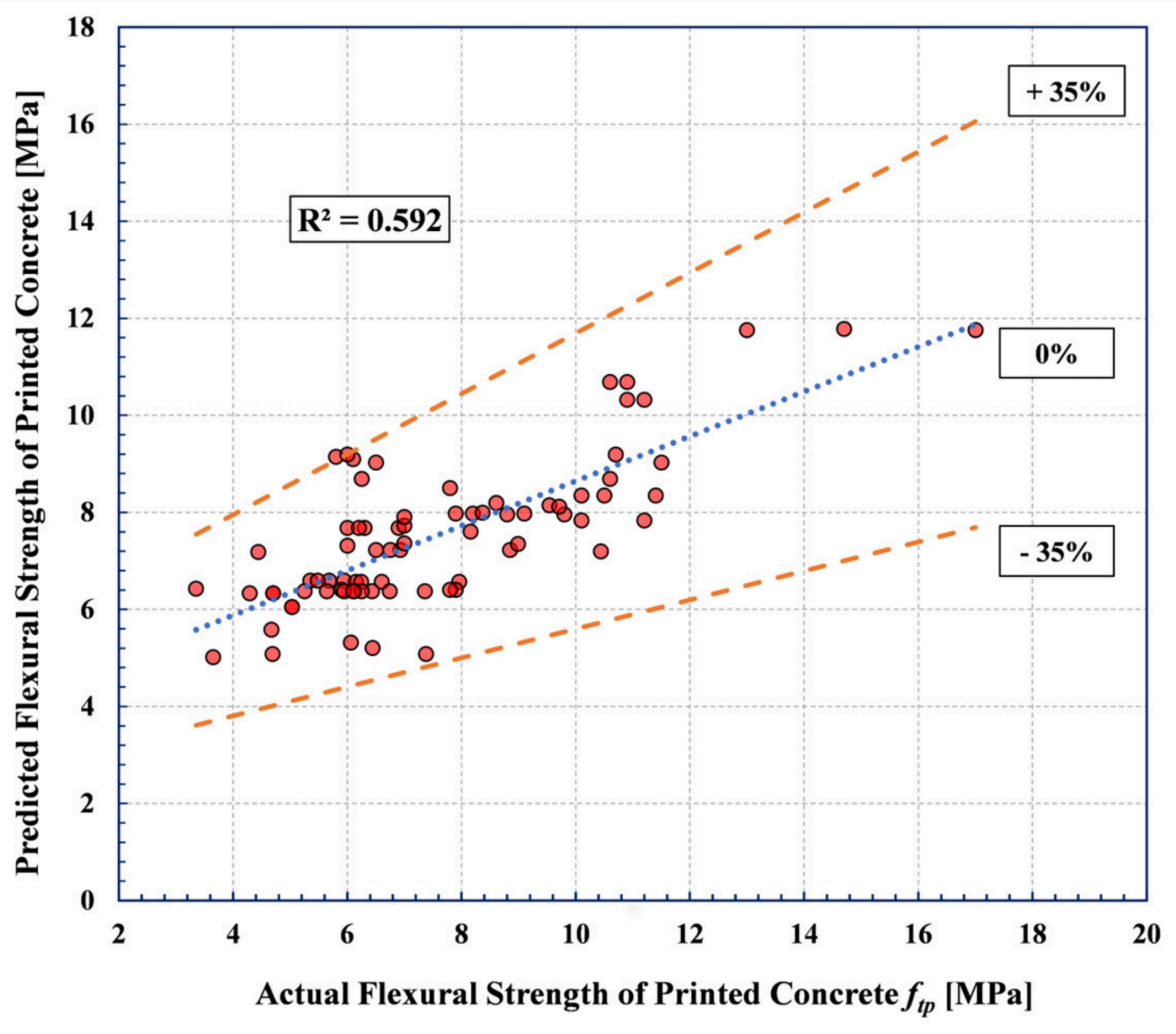
Relation between the actual flexural strength of the printed specimens and the predicted strength.

By mapping the results of Figure 7 onto Equation (9), the estimated flexural strength of the printed samples can be written as follows:

(10) ftp=0.522 fb23

(11) ftKp=0.338 fb23

where $f_{tp}$ is the mean flexural strength of the printed concrete elements, $f_{b}$ is the compressive strength of the casted samples, and $f_{tKp}$ is the characteristic flexural strength of the printed concrete elements. Figure 8 presents the association between the actual flexural strength exhibited by the printed specimens and the strength values anticipated via the statistical analysis method Equation (10).

In the case of the flexural-strength analysis, the initial linear regression between the compressive strength of the casted specimens and the flexural strength of the printed material resulted in an (*R*^2^) value of 0.592. This indicates that only about 59% of the variation in the printed flexural strength could be explained by the cast compressive strength, which is considered relatively weak for developing a reliable predictive relationship [106]. Such a low correlation suggested that the behaviour between these two parameters is not strictly linear and that the dataset required a more advanced statistical treatment to obtain a meaningful design equation. To address this, an extended statistical analysis was performed to investigate whether alternative modelling approaches could better capture the underlying trend.

**Figure 9:**
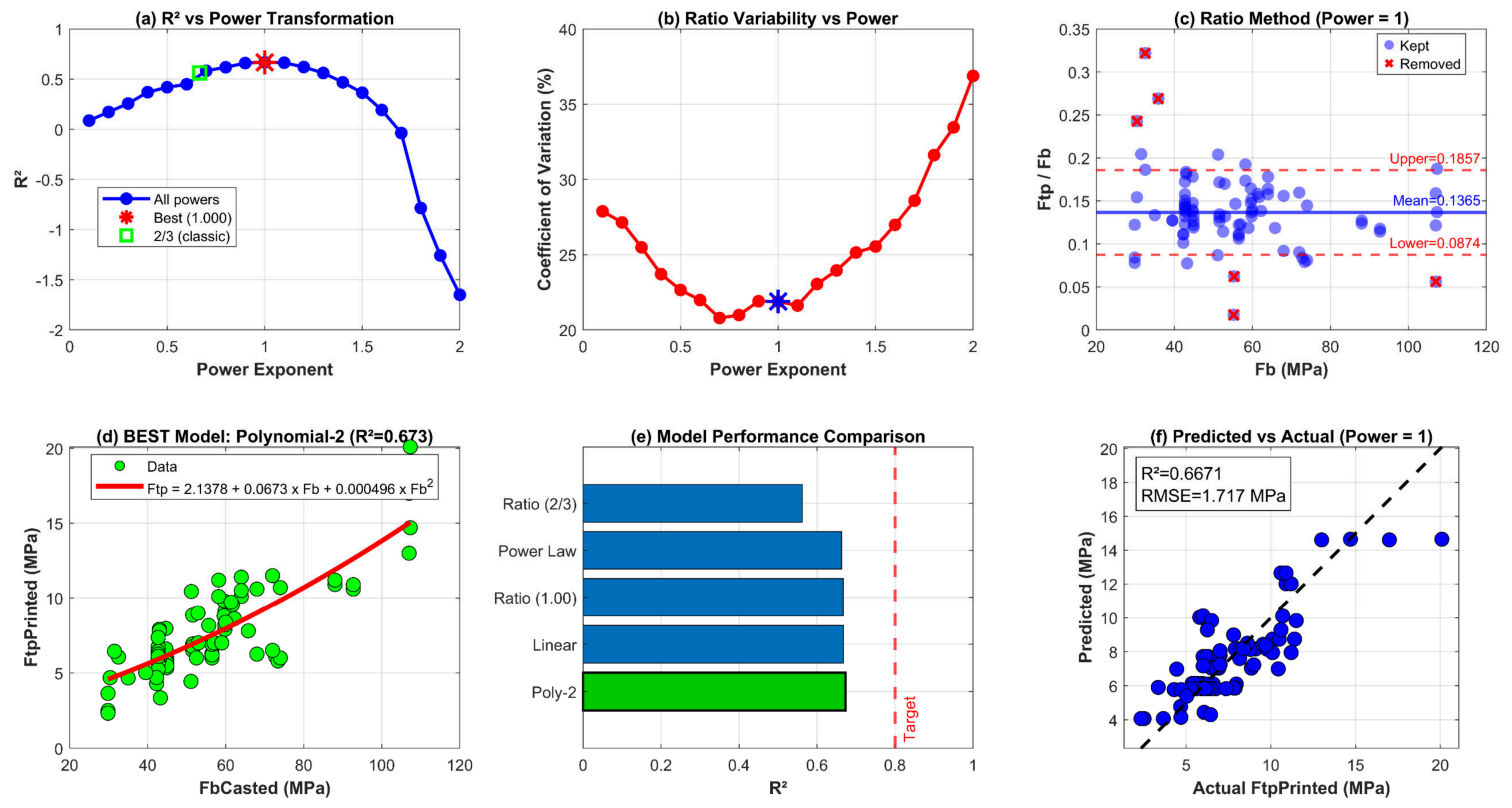
Comprehensive statistical optimisation and performance evaluation of predictive models for the flexural strength of printed concrete.

The limitations of the initial regression were addressed through the expanded statistical evaluation illustrated in Figure 9. As shown in the (*R*^2^) vs. Power Transformation plot (Figure 9a), the correlation significantly improves as the exponent increases from the classical 2/3 value (*R*^2^ *=* 0.592), consistent with fundamental material-strength scaling principles found in Eurocode 2 [110] and ACI 318 [112], toward an optimised value of 1.00. The Ratio Variability analysis (Figure 9b) further confirms that an exponent of 1.00 yields the most stable ratio with a minimised coefficient of variation [106].

While the Model Performance Comparison (Figure 9e) and Table 3 indicate that the second-degree polynomial model achieves the highest mathematical fit (*R*^2^ *=* 0.6732), it was intentionally excluded from the final design framework. This model was rejected because it lacks physical reliability, in structural engineering, a second-degree polynomial can create a downward curve at higher strength ranges, suggesting that concrete becomes weaker as its cast strength increases, a trend that contradicts the established physics of concrete materials [113]. Furthermore, as summarised in Table 2, the difference in (*R^2^*) between the polynomial model and the Optimised Ratio Method (*R*^2^ *=* 0.6671) is negligible. Consequently, following methodological consistency across all direct regression models, the simpler power-law model was selected for the final design equation. This ensures better generalizability, avoids overfitting, and maintains strict compatibility with international design standards. The high reliability of this chosen model is visually verified in the Predicted vs. Actual parity plot (Figure 9f), which demonstrates a strong alignment with experimental results across the entire data range [114].

Based on the optimised ratio where the exponent *n* was identified as 1.0, the final recommended design equation for mean flexural strength ($f_{tp}$) is expressed as:

(12) $$f_{tp}=0.137\;f_{b}$$

To satisfy structural safety requirements, the characteristic flexural strength ($f_{tKp}$) is derived as:

(13) $$f_{tKp}=0.087\;f_{b}$$

### 4.3. Shear Strength

Despite the growing interest in 3DCP and its applications, there is a notable scarcity of published data on shear strength. The limited availability of published data on the shear strength of 3DCP elements is a significant research gap that hinders a comprehensive understanding of its mechanical behaviour. In contrast to the compressive and flexural strength analyses, a similar methodology will be applied to address considerations of shear strength. The relevant reference codes in this context are EC2 and ACI 318-19. In accordance with EC2 and referencing Clause 6.2.2, the equations that characterise the characteristic shear strength are presented below:

(14) vmin=0.035 k32 fck12k=1 +200d ≤2.0fvk=(vmin + k1 σcp) γc

where $v_{min}$ is the minimum design shear strength in N/mm^2^, k is the depth factor estimated to be equal to 2.0 since all the tested specimens are less than 200 mm, $f_{ck}$ is the characteristic compressive strength in N/mm^2^, $f_{vk}$ is the characteristic shear strength in N/mm^2^, and $\sigma_{cp}$ is the axial strength estimated to be equal to 0 because there was no axial force applied during the test. $\gamma_{c}$ is the safety factor, and it is equal to 1.5. Therefore, the characteristic shear strength in terms of the characteristic compressive strength can be rewritten as follows:

(15) fvk=0.149 fck12

ACI 318-19 outlines in Clause R22.5.5 (Table 22.5.5.1) the equation of the characteristic shear strength as follows:

(16) fvk=2 λ fc′ 12+ Nu6Ag  (PSI unit)

Equation (16) is in the PSI unit, and the value of the axial force $N_{u}$ is equal to 0 because there was no axial force applied during the test. The value of the size effect modification factor λ is equal to 1.0. Accordingly, Equation (17) can be written in MPa as follows:

(17) fvk=0.166 fc′ 12 (MPa unit)

**Figure 10:**
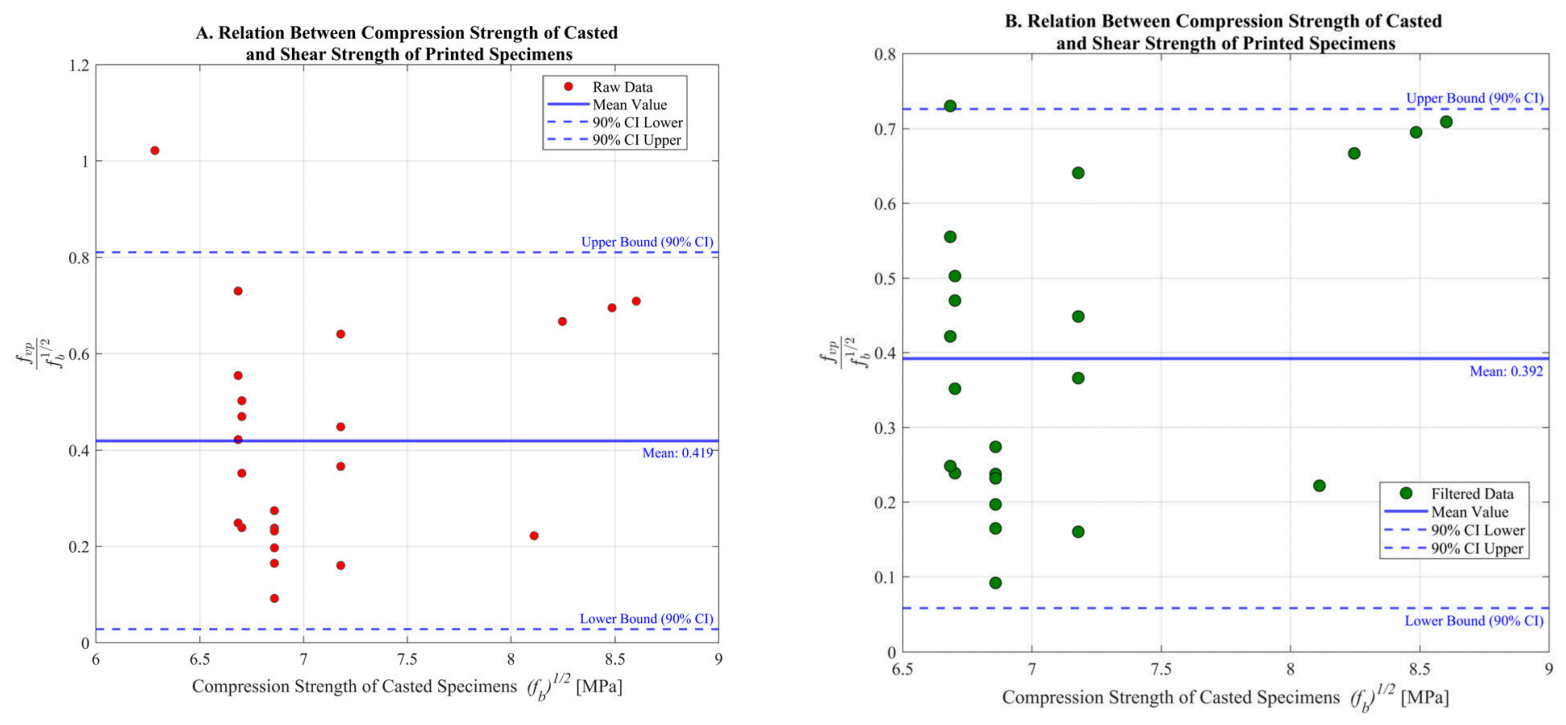
Statistical analysis of shear strength data: (**A**) All collected data, (**B**) Filtered data.

Similarly, the equation derived from the statistical analysis will adhere to the formulation specified in EC2 and ACI 318. It can be reformulated as follows:

(18) fvkp=RLower fb12

where $f_{vkp}$ is the characteristic shear strength of the printed concrete in N/mm^2^, $R_{Lower}$ is the lower bound value estimated from the statistical analysis of the collected data, and $f_{b}$ is the compressive strength of the mortar in N/mm^2^. Figure 10 presents the statistical analysis results conducted on the collected and filtered shear strength data. By mapping the analysis of Figure 10 onto Equation (18), the predicted shear strength of the printed samples can be developed as follows:

(19) fvp=0.392 fb12

(20) fvkp=0.058 fb12

where $f_{vp}$ is the mean shear strength of the printed concrete elements, $f_{b}$ is the compressive strength of the casted samples, and $f_{vkp}$ is the characteristic shear strength of the printed concrete elements.

**Figure 11:**
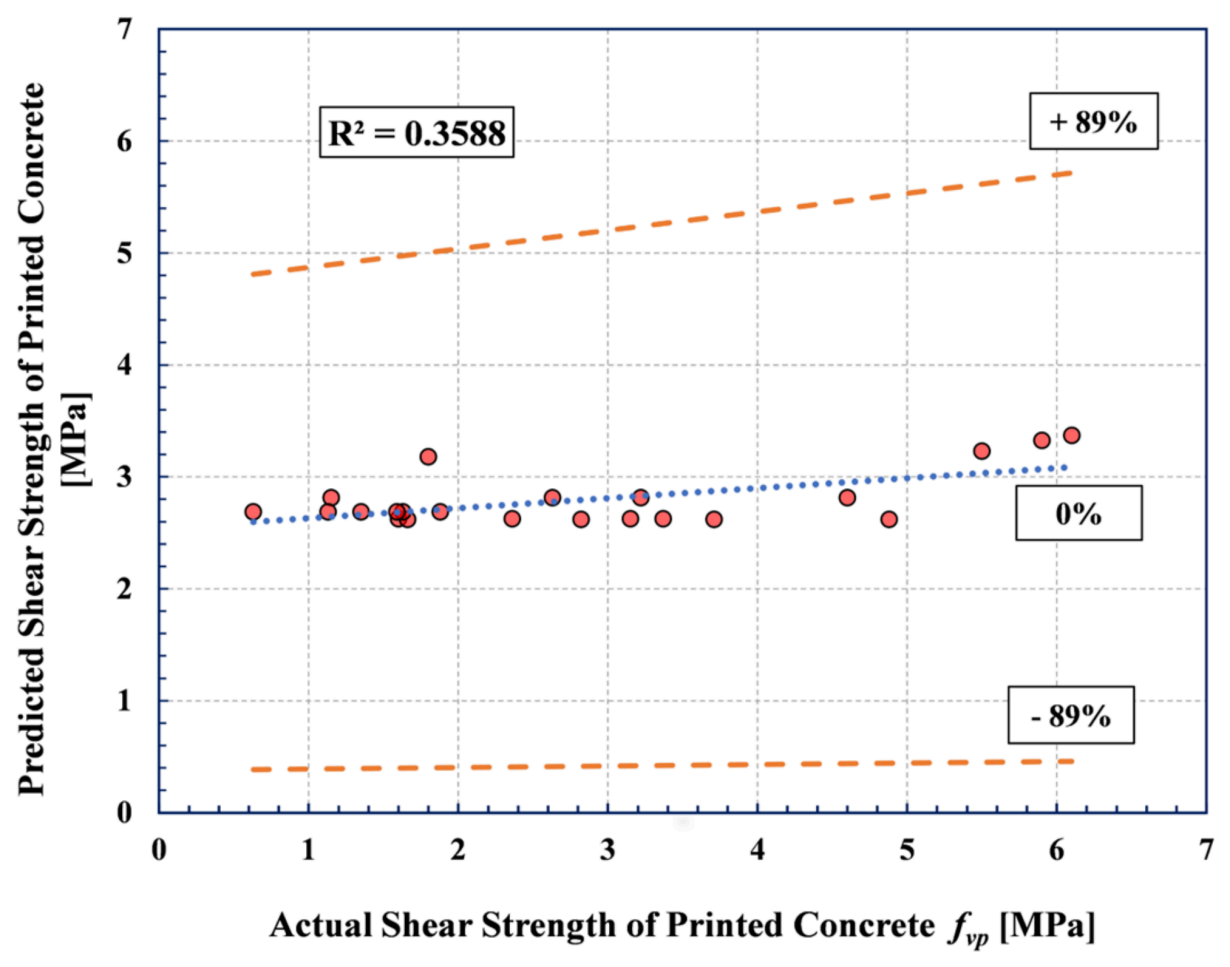
Relation between actual shear strength of the printed specimens and the predicted strength.

Additionally, Figure 11 illustrates the relationship between the actual shear strength values observed in the printed specimens and the corresponding shear strength values predicted by statistical analysis (Equation (19)). When the (R^2^) value in the statistical analysis between the actual and predicted 3DCP shear strength is equal to 0.3588, it means that approximately 35.9% of the variability in the actual shear strength values can be explained by the predicted values obtained from the statistical model [115]. This may be attributed to the scarcity of data, which limits the ability of the model to accurately capture the relationship between the actual and predicted shear strength values. It highlights the importance of acquiring more comprehensive and diverse data to improve the accuracy and reliability of the statistical model for predicting 3DCP shear strength. In this study, the low R^2^ causes a notable coefficient value of 0.058 in the derived Equation (20) compared to the coefficients of 0.166 observed in the case of ACI 318 (Equation (17)) and 0.149 in the case of EC2 (Equation (15)). This finding underscores the limited capacity of 3DCP structures to effectively withstand shear stresses, primarily due to their weak interlocking between the printed layers [116]. By exploring innovative techniques, materials, and construction methodologies, it is possible to enhance the interlocking mechanisms and improve the shear resistance capabilities of 3DCP structures [117]. Advancing the understanding of interlayer bonding mechanisms and optimising shear strength resistance in 3DCP structures is crucial for ensuring their structural integrity and reliability in diverse practical applications [118].

To enhance the empirical predictive capability of the design framework, a multi-stage Enhanced Ratio Method Analysis was performed on a refined dataset (*N* = 22) following a 90% Confidence Interval (CI) filtering process. The comprehensive performance of the various statistical models tested is summarised in Table 3. The analysis first evaluated the Classic Ratio Method, which utilises the 1/2 power law common in traditional standards such as ACI 318 [112] and Eurocode 2 [110]. As shown in Table 3, this conventional approach yielded a significantly poor fit for 3DCP (*R*^2^ = 0.3588, *RMSE* = 1.4758 MPa), confirming that the square-root relationship developed for isotropic monolithic concrete fails to account for the anisotropic shear planes inherent in 3DCP.

**Figure 12:**
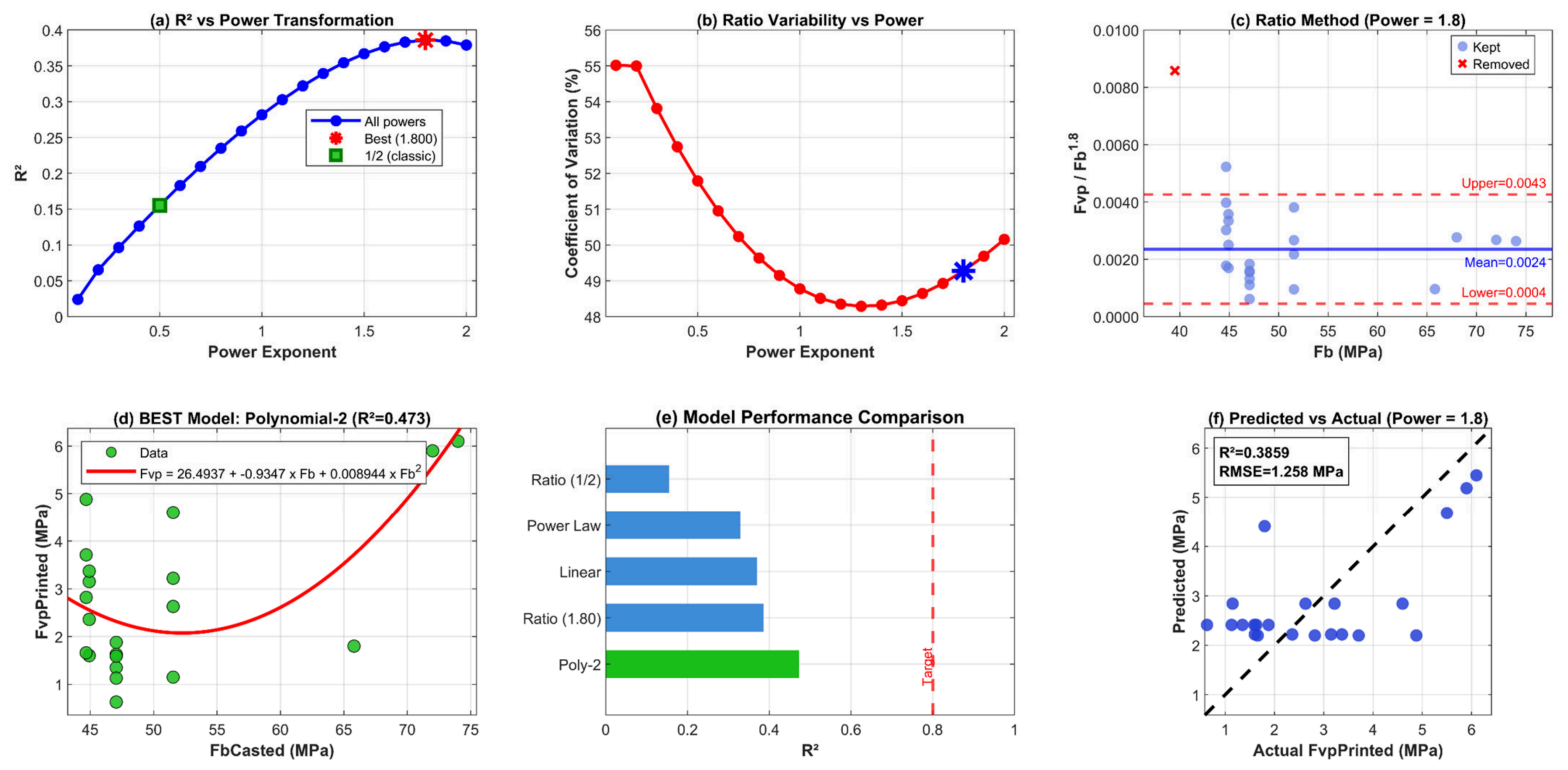
Comprehensive statistical optimisation and performance evaluation of predictive models for the shear strength of printed concrete.

In response to this deficiency, an Optimised Power Ratio was investigated across an exponent range of 0.10 to 2.00. As illustrated in Figure 12, the model achieved peak convergence at an exponent of *n* = 1.80, yielding a superior correlation of R^2^ = 0.3859 and a reduced RMSE of 1.2583 MPa. This shift from the standard *n* = 0.5 toward a significantly higher exponent suggests that shear resistance in 3DCP is disproportionately dependent on the material grade. This is likely due to the fact that higher-strength mixes facilitate better interlaminar compaction and interfacial bond efficiency, which are the primary drivers of shear capacity in layered elements.

While the second-degree polynomial model mathematically achieved the highest correlation (*R*^2^ = 0.4731, *RMSE* = 1.1655 MPa), it was intentionally excluded from the final design framework for the same reason outlined in the flexural strength prediction in Section 4.2. Consequently, the Optimised Ratio Method (*n* = 1.80) was adopted as the most robust basis for the mean shear strength (f_vp_) equation:

(21) $$f_{vp}=0.0024\;{f_{b}}^{1.8}$$

To satisfy structural safety requirements, the characteristic shear strength ($f_{vKp}$) is derived as:

(22) $$f_{vkp}=0.0004\;{f_{b}}^{1.8}$$

## 5. Machine Learning Analysis

Unlike traditional regression-based analytical approaches, machine learning models can capture multivariate interactions without the need to predefine functional relationships. This capability is particularly relevant for 3DCP materials, where strength development is influenced not only by mixture proportions but also by process-induced parameters such as layer geometry and printing speed. Previous studies have demonstrated the effectiveness of ML algorithms in predicting concrete mechanical properties with high accuracy [73,74,119].

The ML models were developed using the comprehensive database compiled from the literature and organised in the table provided in Table S1. The target output variables predicted by the machine learning models were printed compressive strength and printed flexural strength. To ensure a direct and equivalent comparison with the derived statistical design equations, the machine learning models were trained using a single input feature. For all models developed in this study, the sole input variable was the cast compressive strength, which was used to predict either the printed compressive or printed flexural strength independently. Where multiple curing ages were reported, 28-day strength values were prioritised to maintain consistency across the database and reduce variability associated with early-age behaviour. Prior to model training, the dataset underwent systematic pre-processing. Duplicate entries were removed, and incomplete records lacking essential mechanical or compositional data were excluded. Only parameters consistently reported across enough studies were retained to ensure statistical robustness. All numerical features were normalised using Min–Max scaling to improve convergence behaviour and prevent dominance of high-magnitude variables during model training [75]. The dataset was randomly divided into training and testing subsets to evaluate the generalisation capability of the developed machine learning models and minimise overfitting. The models were developed using the complete initial datasets, comprising 116 paired observations for printed compressive strength prediction and 86 paired observations for printed flexural strength prediction. As previously clarified, a single input feature (cast compressive strength) was employed for all predictions. A 90/10 training-to-testing split ratio was adopted, resulting in 104 training and 12 testing observations for compressive strength prediction, and 77 training and 9 testing observations for flexural strength prediction. To enhance the reliability of model evaluation, 10-fold cross-validation was performed within the training subsets, while the independent testing subsets were reserved for the final evaluation of predictive performance.

**Figure 13:**
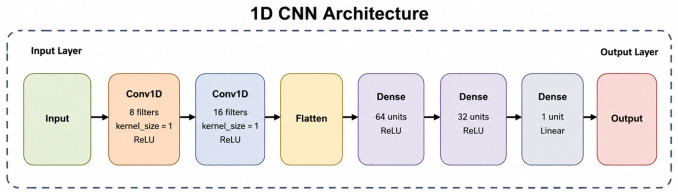
Proposed 1D Convolutional Neural Network (CNN) architecture.

Four supervised learning algorithms were implemented in this study: Linear Regression (LR), Random Forest (RF), Multilayer Perceptron (MLP), and one-dimensional Convolutional Neural Network (1D CNN). Linear Regression is a foundational statistical approach that models the linear relationship between independent input features and a continuous target variable, serving as a highly interpretable baseline for comparative performance analysis [102]. Random Forest is an ensemble tree-based method that improves predictive performance through bootstrap aggregation and random feature selection [120]. It is particularly robust when modelling nonlinear material behaviour. The Multilayer Perceptron, a feedforward artificial neural network with nonlinear activation functions, was employed to capture complex, nonlinear relationships between the cast and printed strengths [121]. Additionally, a lightweight one-dimensional CNN architecture was designed to automatically extract hidden feature patterns from structured numerical data, as deep learning models have shown strong capability in materials modelling applications [122]. The proposed lightweight 1D-CNN presented in Figure 13 consists of two one-dimensional convolutional layers followed by three fully connected (FC) layers. The convolutional layers are responsible for extracting discriminative feature representations from the input data. The first convolutional layer employs 8 filters, while the second convolutional layer utilises 16 filters. The extracted features are flattened and passed through three FC layers containing 64 neurons, 32 neurons, and 1 neuron, respectively. Rectified Linear Unit (ReLU) activation is used in all hidden layers, whereas a linear activation function is employed in the output layer to perform regression. The model is trained using the Adam optimiser, and the mean squared error (MSE) is adopted as the loss function. Owing to its small number of layers and parameters, the proposed architecture is computationally lightweight and suitable for efficient regression-based prediction tasks.

Model performance was evaluated using the R^2^, MAE, and RMSE. These metrics are widely adopted for assessing predictive accuracy in concrete strength modelling [121]. Higher R^2^ values and lower MAE and RMSE values indicate improved predictive capability. Finally, the ML results were compared with the optimised power-law statistical relationships developed earlier in this study. While machine learning models demonstrated strong predictive performance due to their ability to account for multiple interacting variables simultaneously, the analytical models offered greater interpretability and simplicity for practical engineering applications. Therefore, the ML framework was primarily employed as a validation and benchmarking tool to confirm the robustness of the proposed statistical relationships.

It should be noted that, although compressive and flexural strengths were analysed using the machine learning framework, the shear strength of 3DCP was not included in the ML modelling. This is primarily due to the insufficient amount of reliable and consistently reported shear strength data available in the published literature. Compared with compressive and flexural tests, shear testing of 3DCP is less commonly conducted and lacks standardised experimental procedures, resulting in a limited dataset that is not adequate for training and validating data-driven models. Since machine learning algorithms require sufficiently large and representative datasets to ensure reliable generalisation and avoid overfitting [74,75], shear strength prediction was excluded from the ML analysis. Nevertheless, shear behaviour was investigated using the statistical modelling approach presented in the previous sections.

The statistical performance metrics detailed in Table 4, specifically *R*^2^, *MAE*, and *RMSE*, demonstrate the predictive superiority of the proposed 1D Convolutional Neural Network (1D CNN). Achieving the highest (*R*^2^) values and lowest error margins for both compressive and flexural strength datasets, the 1D CNN emerges as the most robust architecture for this study. This success highlights the architectural advantages of the 1D CNN. Unlike traditional regression or tree-based models, which often rely on manually selected input features, the 1D CNN uses hierarchical feature extraction to autonomously detect complex, non-linear patterns within the sequential, layer-by-layer deposition data inherent to 3DCP. These results establish the 1D CNN as a highly effective, end-to-end framework for predicting material properties, offering a precise and scalable method for structural analysis in 3D concrete printing.

Table 5 provides a comparative overview of the 3DCP strength predictions generated by the proposed 1D CNN model, contrasted against the analytical outputs of the power-law equations. This framework quantifies the predictive fidelity of each modelling strategy under standardised, unified conditions. To ensure practical engineering relevance and circumvent the risks associated with extrapolating beyond the training data, all predictions were confined to a casted compressive strength range of 30–80 MPa, a domain representing the standard structural-grade threshold in the current 3DCP literature. A 5 MPa increment was adopted for this analysis to provide sufficient granularity for observing non-linear behavioural trends in strength development.

**Figure 14:**
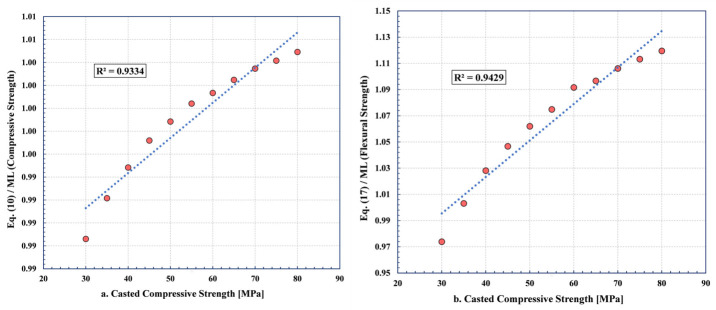
Correlation between 1D CNN model predictions and derived equations for (**a**) compressive and (**b**) flexural strength.

Figure 14 compares the 1D CNN predictions with the values generated by the derived equations for (a) compressive strength and (b) flexural strength. The proposed equations exhibit robust agreement with the 1D CNN model, with relative deviations predominantly remaining within a 10% threshold across the dataset. While a notable divergence appears in the flexural strength data at magnitudes exceeding 70 MPa (Figure 14a), this discrepancy is practically negligible, as concrete strengths of this range are rarely utilised in conventional structural applications. The agreement between the machine learning results and the statistical equations proposed in this study demonstrates the validity and reliability of the developed equations.

## 6. Design Strength

Concrete characteristic strength, design strength, and safety factors are crucial in ensuring the structural integrity and safety of concrete elements. The safety factor accounts for uncertainties and variations in material properties, workmanship, and environmental conditions. In EC6 [105] and EC2 [110], a common safety factor value of 1.5 is often used in the design process. The design strength is obtained by dividing the characteristic strength by the safety factor. ACI 318 [111] follows a similar approach by incorporating a strength reduction factor denoted as φ. The characteristic strength ($f_{c}^{\prime }$) is multiplied by the strength reduction factor of 0.65 to obtain the design strength, which is broadly equivalent to the partial safety factor of 1.5 adopted in the Eurocodes.

In this study, focusing on 3DCP structures, a safety factor of 1.5 will be employed to ensure the safety of the design. As a result of the current work, Table 6 serves as a reference for practitioners engaged in the field of 3DCP, aiding them in determining the design strength values that conform to safety standards and meet the required structural performance criteria. By utilising these proposed design strength equations, engineers can enhance the safety and reliability of 3DCP structures, contributing to the further advancement and widespread adoption of this innovative construction technology.

## 7. Conclusions

This research establishes a valuable computational framework that addresses the critical lack of standardised design procedures for 3DCP. By synthesising analytical power-law modelling with advanced Machine Learning analysis, the study offers a dual-faceted methodology capable of translating experimental mechanical properties into reliable, actionable parameters for structural engineering.

The comparative analysis presented in Figure 14 demonstrates good agreement between the derived preliminary equations and the high-fidelity machine learning predictions. Furthermore, Table 5 provides preliminary reference values derived from the proposed analytical approaches based on the currently available experimental database. Quantitatively, the statistical analysis established mean strength ratios of 0.868 and 0.137 for printed compressive and flexural strengths, respectively, relative to cast compressive strength. Using the safety factor adopted in this study, the resulting preliminary design strength ratios were 0.391 for compressive strength and 0.058 for flexural strength. For shear strength, the analysis indicated that the conventional square-root relationship showed limited predictive capability for interlayer behaviour, leading to the adoption of an optimised power-law relationship with an exponent of 1.8. Among the evaluated machine learning algorithms, the 1D CNN model achieved the highest predictive performance within the available dataset, with R^2^ values of 0.9689 and 0.7874, MAE values of 4.333 MPa and 1.099 MPa, and RMSE values of 4.932 MPa and 1.275 MPa for compressive and flexural strength predictions, respectively.

This study presents preliminary strength estimation relationships, summarised in Table 6, intended to support the initial assessment and feasibility evaluation of hardened 3DCP structures under different loading conditions. These relationships are not intended to replace comprehensive experimental validation but rather provide a data-driven approach for preliminary evaluation based on the currently available evidence.

Despite the correlations identified in this study, several limitations should be acknowledged. First, the predictive relationships are inherently constrained by the size and variability of the available literature database, particularly for shear strength data, where limited experimental observations were available. Second, the derived equations represent generalised preliminary relationships and do not explicitly incorporate individual process parameters, such as printing speed, nozzle geometry, layer time interval, or proprietary mix formulations. Finally, the machine learning models were developed within the range of parameters available in the current literature, highlighting the need for larger experimental campaigns and independent validation studies to further improve and verify the applicability of the proposed relationships.

Looking forward, this study provides the foundation for the next phase of 3DCP standardisation. Future research should focus on expanding the available databases, particularly for shear strength, and incorporating wider variations in material compositions, printing parameters, environmental conditions, and real-time monitoring data. Such developments will enhance the reliability and applicability of predictive models and contribute towards the future establishment of standardised structural design approaches for 3DCP.

Ultimately, the predictive models and preliminary design equations developed in this research provide a practical, data-driven framework for estimating the mechanical properties of 3D-printed concrete, thereby supporting ongoing efforts towards the development of standardised structural design guidelines.

## Figures and Tables

**Table 2:** Summary of Statistical Models Developed for Predicting Flexural Strength of Printed Concrete.

Method	Model Type/ Transformation	Best-Fit Equation	R^2^	RMSE [MPa]
Classic Ratio (2/3 Power)	ftp/fb23	ftp=0.522 fb23	0.5662	1.6725
Optimised Ratio Method	$f_{tp}/{f_{b}}^{n}$	Best exponent *n* = 1.0	0.6671	1.7166
Linear Regression	$f_{tp}=a+b\;f_{b}$	$f_{tp}=0.152+0.133\;f_{b}$	0.6675	1.7156
Power-Law Regression	$f_{tp}=a\;{f_{b}}^{b}$	$f_{tp}=0.145\;{f_{b}}^{0.98}$	0.6629	1.7275
Polynomial Regression (2nd Degree)	$f_{tp}=a+b\;f_{b}+c\;{f_{b}}^{2}$	$f_{tp}=2.138+0.067\;f_{b}+0.0005\;{f_{b}}^{2}$	0.6732	1.7010

**Table 3:** Summary of Statistical Models Developed for Predicting Shear Strength of Printed Concrete.

Method	Model Type/ Transformation	Best-Fit Equation	R^2^	RMSE [MPa]
Classic Ratio (1/2 Power)	fvp/fb12	fvp=0.392 fb12	0.3588	1.4758
Optimized Ratio Method	$f_{vp}/{f_{b}}^{n}$	Best exponent *n* = 1.8 $f_{vp}=0.0024\;{f_{b}}^{1.8}$	0.3859	1.2583
Linear Regression	$f_{vp}=a+b\;f_{b}$	$f_{vp}=-2.5628+0.1057\;f_{b}$	0.3699	1.2746
Polynomial Regression (2nd Degree)	$f_{vp}=a+b\;f_{b}+c\;{f_{b}}^{2}$	$f_{vp}=26.4937-0.9347\;f_{b}+0.008944\;{f_{b}}^{2}$	0.4731	1.1655

**Table 4:** Summary of ML analysis.

Model	R^2^	MAE	RMSE
Compressive strength			
Linear Regression	0.8783	7.8120	10.6254
Random Forest	0.9116	5.3696	7.0358
Multilayer Perceptron	0.8871	8.2709	10.2342
1D CNN	0.9689	4.333	4.9322
Flexural strength			
Linear Regression	0.6862	1.3853	1.5484
Random Forest	0.6529	1.6317	2.0102
Multilayer Perceptron	0.7786	1.12	1.3125
1D CNN	0.7874	1.0992	1.2746

**Table 5:** Predicted 3DCP strengths using the derived equations from the statistical analysis and 1D CNN model.

Casted Compressive Strength [MPa]	Predicted Compressive Strength by ML (1D CNN) [MPa]	Predicted Flexural Strength by ML (1D CNN) [MPa]	Predicted Compressive Strength Equation (5) [MPa]	Predicted Flexural Strength Equation (12) [MPa]	Predicted Shear Strength Equation (21) [MPa]	Characteristic Compressive Strength Equation (6) [MPa]	Characteristic Flexural Strength Equation (13) [MPa]	Characteristic Shear Strength Equation (22) [MPa]
30	26.34	4.22	26.04	4.11	1.094	17.58	2.61	0.182
35	30.62	4.78	30.38	4.795	1.444	20.51	3.045	0.241
40	34.90	5.33	34.72	5.48	1.836	23.44	3.48	0.306
45	39.17	5.89	39.06	6.165	2.270	26.37	3.915	0.378
50	43.45	6.45	43.4	6.85	2.744	29.3	4.35	0.457
55	47.72	7.01	47.74	7.535	3.257	32.23	4.785	0.543
60	52.01	7.53	52.08	8.22	3.810	35.16	5.22	0.635
65	56.28	8.12	56.42	8.905	4.400	38.09	5.655	0.733
70	60.55	8.67	60.76	9.59	5.028	41.02	6.09	0.838
75	64.83	9.23	65.1	10.275	5.693	43.95	6.525	0.949
80	69.10	9.79	69.44	10.96	6.394	46.88	6.96	1.066

**Table 6:** Design strength equations.

No	Strength	Characteristic Strength Equation	Design Strength Equation
1	Compressive Strength	$f_{ckp}=0.586\;f_{b}$ (Equation (6))	$f_{cdp}=0.391\;f_{b}$
2	Flexural Strength	$f_{tKp}=0.087\;f_{b}$ (Equation (13))	$f_{tdp}=0.058\;f_{b}$
3	Shear Strength	$f_{vkp}=0.0004\;{f_{b}}^{1.8}$ (Equation (22))	$f_{vdp}=0.00026\;{f_{b}}^{1.8}$

Note: $f_{b}$ is the compressive strength of the casted samples.

## Data Availability

The original contributions presented in this study are included in the article/Supplementary Materials. Further inquiries can be directed to the corresponding author.

## Supplementary Materials

The following supporting information can be downloaded at: https://www.mdpi.com/article/10.3390/ma19153358/s1, Table S1: Summary of Collected Data from 3D Concrete Printing Literature.
