# Supplementary material for: Optimised Machine Learning and Statistical Modelling for Predicting the Design Strengths of Hardened 3D Printed Concrete
Source: Materials (Basel). 2026 Aug 6;19(15):3358. doi: 10.3390/ma19153358 (PMC13467685; doi:10.3390/ma19153358)
Supplement: Supplementary file 1 [file materials-19-03358-s001.zip › materials-4465941-supplementary.pdf]

Table S1: Summary of Collected Data from 3D Concrete Printing Literature

No.	Sample Specifications (Mix Design)	Material Properties: W/B	Flow [mm]	IST [min]	Cement [kg/m^3]	Open Time [min]	Sand/ Binder	SCM / Cement: FA/C	SF/C	LP/C	CSA/C	NA/C	HPMC/C	Slag/C	Admixtures / Cement: SP/C	Acc/Ret/C	PE Fiber/C	Admixture Type	Mechanical Properties: Cast Specimens [MPa] – 28 days: Comp.	Tension	Shear	3DCP Specimens [MPa]: Comp.	Tension	Shear	Poisson Ratio	Young's Mod. [MPa]	Printing Parameters: Temp [°C]	Curing Conditions	Layer W. [mm]	Layer H. [mm]	Speed [mm/s]	Nozzle Diam. [mm]	Ref.
1	Cement proportion 1, sand 1.05, water 0.4, Basalt Fiber (Vol%) 2.5, 5, 7.5, 10 - Superplasticizer 0.25 - 0.56 and accelerator 6	0.4	-	162	-	-	1.05	0	0	0	0	0	0	0	-	-	0	-	55.65	7.86	-	44.48	8.16	-	-	-	25	Wet curing	-	7	40 to 70	15	[37]
		0.4	-	162	-	-	1.05	0	0	0	0	0	0	0	-	-	0	-	61.72	8.5	-	44.94	9.54	-	-	-			-				
		0.4	-	162	-	-	1.05	0	0	0	0	0	0	0	-	-	0	-	61.37	8.79	-	51.54	9.71	-	-	-			-				
		0.4	-	162	-	-	1.05	0	0	0	0	0	0	0	-	-	0	-	62.23	9.04	-	52.59	8.61	-	-	-			-				
		0.4	-	162	-	-	1.05	0	0	0	0	0	0	0	-	-	0	-	59.99	8.11	-	49.64	8.37	-	-	-			-				
2	UHPC 1, Fine sand 0.5, Water 0.19, Early strength agent 0.014, Cellulose thickener 0.0016, HRWRA 0.01	0.19	-	-	-	-	-	0	0	0	0	0	0	0	0	0	0	-	43.26	3.92	-	34.21	3.35	-	-	-	-	-	-	10, 15, 30, 45	-	-	[38]
3	OPC 0.6, FA 0.3, SF 0.1, NC/B 0.005, SS/B 0.2, Water 0.3, PE VD% 1	0.3	-	-	-	-	0.2	0.3	0.1	0	0	0	0	0	0	0	0	-	52.9	13.76	-	64.81	8.99	-	0.2	2.00E+04	20	Curing in a curing chamber	-	5, 10, 15	150	30 x 5	[39]
4	Cement 299.2 kg/m3, Limestone powder 81.6, Calcined clay 163.2, Fly ash 652.8, Silica Sand 435.2, Water 299.2, SP 6.5, VMA 1.8, PVA fiber 26	0.25	130	-	299.2	60	0.8	1.2	0	0.15	0	0	0	0.3	0.0055	0.0015	0	Polycarboxylate ether (PCE)-based	32	20	-	36	25	-	-	-	20	-	-	7 - 7.2	50	15	[40]
5	Cement 0.72, GGBS 0.2, Alccofine 1203 0.08, M-Sand 1, W/C 0.35, SP 0.002	0.35	156	65	720	25	1	0	0	0	0	0	0	0.278	0.00278	0	0	Polycarboxylate ether (PCE)-based	31.5	6.63	-	22.7	6.44	-	-	-	-	Wet curing	-	-	20	25 × 12 (width X height)	[41]
6	MFC_S	0.4	145	290	625	127	0.6	0	0	0	0	0	0	0	0.00215	0	0	Polycarboxylate ether (PCE)-based	57	8.5	-	24	7	-	-	-	20	After 28 days of curing in water, three specimens from cast mortars and six 3DCP specimens in accordance with ASTM C624	15	5	50	13	[42]
	MFC_P	0.4	147	290	625	123	0.6	0	0	0	0	0	0	0	0.00215	0	0	Polycarboxylate ether (PCE)-based	59	8.5	-	24	7	-	-	-	-		-	-	-	-	
	HPMC	0.4	133	365	625	128	0.6	0	0	0	0	0	0	0	0.00215	0	0	Polycarboxylate ether (PCE)-based	53	8.5	-	24	7	-	-	-	-		-	-	-	-	
7	Control	0.26	214	-	-	-	1	0	0	0	0	0	0	0	0.002	0.0003	0	Polycarboxylate-based, PC	59.6	12.1	-	60.4	9.8	-	-	-	23	-	-	-	25	40 x 20	[43]
	Control	0.26	214	-	-	-	1	0	0	0	0	0	0	0	0.002	0.0003	0	Polycarboxylate-based, PC	59.6	12.1	-	51.2	8.8	-	-	-	-	-	-	-			
	Control	0.26	214	-	-	-	1	0	0	0	0	0	0	0	0.002	0.0003	0	Polycarboxylate-based, PC	59.6	-	-	55.8	-	-	-	-	-	-	-	-			
	PP-05	0.26	174	-	-	-	1	0	0	0	0	0	0	0	0.002	0.0003	0	Polycarboxylate-based, PC	58.2	13.7	-	53.3	11.2	-	-	-	-	-	-	-			
	PP-05	0.26	174	-	-	-	1	0	0	0	0	0	0	0	0.002	0.0003	0	Polycarboxylate-based, PC	58.2	13.7	-	71.7	10.1	-	-	-	-	-	-	-			
	PP-05	0.26	174	-	-	-	1	0	0	0	0	0	0	0	0.002	0.0003	0	Polycarboxylate-based, PC	58.2	-	-	70.4	-	-	-	-	-	-	-	-			
	ST-05	0.26	205	-	-	-	1	0	0	0	0	0	0	0	0.002	0.0003	0	Polycarboxylate-based, PC	88	13.1	-	77.4	10.9	-	-	-	-	-	-	-			
	ST-05	0.26	205	-	-	-	1	0	0	0	0	0	0	0	0.002	0.0003	0	Polycarboxylate-based, PC	88	13.1	-	54.4	11.2	-	-	-	-	-	-	-			
	ST-05	0.26	205	-	-	-	1	0	0	0	0	0	0	0	0.002	0.0003	0	Polycarboxylate-based, PC	88	-	-	77.8	-	-	-	-	-	-	-	-			
	ST-1	0.26	201	-	-	-	1	0	0	0	0	0	0	0	0.002	0.0003	0	Polycarboxylate-based, PC	92.7	15.2	-	92.3	10.6	-	-	-	-	-	-	-			
	ST-1	0.26	201	-	-	-	1	0	0	0	0	0	0	0	0.002	0.0003	0	Polycarboxylate-based, PC	92.7	15.2	-	73.8	10.9	-	-	-	-	-	-	-			
	ST-1	0.26	201	-	-	-	1	0	0	0	0	0	0	0	0.002	0.0003	0	Polycarboxylate-based, PC	92.7	-	-	91.8	-	-	-	-	-	-	-	-			
	ST-2	0.26	188	-	-	-	1	0	0	0	0	0	0	0	0.002	0.0003	0	Polycarboxylate-based, PC	107.3	27.8	-	114.2	14.7	-	-	-	-	-	-	-			
	ST-2	0.26	188	-	-	-	1	0	0	0	0	0	0	0	0.002	0.0003	0	Polycarboxylate-based, PC	107.3	27.8	-	93.1	20.1	-	-	-	-	-	-	-			
	ST-2	0.26	188	-	-	-	1	0	0	0	0	0	0	0	0.002	0.0003	-	Polycarboxylate-based, PC	107.3	-	-	101.3	-	-	-	-	-	-	-	-			
8	-	0.3	210	-	382.5	65	0.29	1.18	0.34	0.23	0	0	0	0.69	0	0	0	-	29.84	2.93	-	20.98	2.51	-	0.2	1.86E+04	-	-	-	-	60	20	[44]
	-	0.3	210	-	382.5	65	0.29	1.18	0.34	0.23	0	0	0	0.69	0	0	0	-	29.84	2.93	-	27.44	3.65	-			-	-	-	-			
	-	0.3	210	-	382.5	65	0.29	1.18	0.34	0.23	0	0	0	0.69	0	0	0	-	29.84	2.93	-	29.84	2.33	-			-	-	-	-			
9	-	0.58	-	-	424	160	3	0	0	0	0	0	0	0	0.0008	0.01	0	Polycarboxylate ethers	36	-	-	24	-	-	-	-	20	-	55	15	70	35	[45]
10	(CEM I 52.5 N) and CEM II 52.5 N Portland cement (PC), with a relative density (RD) of 3.14 and adhering to BS EN 197-1 - FA-FRPC	0.31	150.5	160	521.4	160	1.61	0.31	0.155	0	0	0	0	0	0.01	0.003	0	-	32.53	6.66	-	32.52	10.47	-	-	-	23	Specimens were made in a climate-controlled room at a temperature	33 - 35	10	-	25	[46]
		0.31	150.5	160	521.4	160	1.61	0.31	0.155	0	0	0	0	0	0.01	0.003	0	-	32.53	6.66	-	36	6.06	-	-	-							
	(CEM I 52.5 N) and CEM II 52.5 N Portland cement (PC), with a relative density (RD) of 3.14 and adhering to BS EN 197-1 - LC3-FRPC	0.45	153	160	381.8	160	1.61	0	0	0.9	0	0	0	0	0.01	0.003	0	-	30.4	7.86	-	25.24	7.38	-	-	-							
		0.45	153	160	381.8	160	1.61	0	0	0.9	0	0	0	0	0.01	0.003	0	-	30.4	7.86	-	30.72	4.69	-	-	-							
11	Normal Weight Concrete	0.32	-	210	703.7	-	0.6	0	0	0	0	0	0	0	0	0.0097	0.0026	Viscosity Modifying Admixture	55.3	3.47	-	57.5	3.44	-	-	-	-	-	100	30	33	100 x 30	[47]
12	Fiber Reinforced Concrete	0.32	-	210	703.7	-	0.6	0	0	0	0	0	0	0	0	0.0097	0.0026	Viscosity Modifying Admixture	51.1	4.31	-	54	4.44	-	-	-	-	-					
13	Components wt% - Cement 55% - FA 20% - SF 15% - GGBS 10% - Sand 130 Water 36% - Superplasticizer 0.23	0.36	-	-	1210	40	1.3	0.2	0.15	0	0	0	0	0.1	0.00029	0	0.00418	Polycarboxylate-based	42.7	11.5	-	55	6.12	-	-	-	-	Steam curing	-	13	120	38 x 14	[48]
		0.36	-	-	1210	40	1.3	0.2	0.15	0	0	0	0	0.1	0.00029	0	0.00418	Polycarboxylate-based	42.7	11.5	-	45.8	6.74	-	-	-	-						
		0.36	-	-	1210	40	1.3	0.2	0.15	0	0	0	0	0.1	0.00029	0	0.00418	Polycarboxylate-based	42.7	11.5	-	50.3	5.94	-	-	-	-						
		0.36	-	-	1210	40	1.3	0.2	0.15	0	0	0	0	0.1	0.00029	0	0.00418	Polycarboxylate-based	42.7	11.5	-	56.7	5.64	-	-	-	-						
		0.36	-	-	1210	40	1.3	0.2	0.15	0	0	0	0	0.1	0.00029	0	0.00418	Polycarboxylate-based	42.7	11.5	-	42.7	6.43	-	-	-	-						
		0.36	-	-	1210	40	1.3	0.2	0.15	0	0	0	0	0.1	0.00029	0	0.00418	Polycarboxylate-based	42.7	11.5	-	45.7	6.25	-	-	-	-						
		0.36	-	-	1210	40	1.3	0.2	0.15	0	0	0	0	0.1	0.00029	0	0.00418	Polycarboxylate-based	42.7	11.5	-	43.6	5.25	-	-	-	-						
		0.36	-	-	1210	40	1.3	0.2	0.15	0	0	0	0	0.1	0.00029	0	0.00418	Polycarboxylate-based	42.7	11.5	-	44.3	6.1	-	-	-	-						
		0.36	-	-	1210	40	1.3	0.2	0.15	0	0	0	0	0.1	0.00029	0	0.00418	Polycarboxylate-based	42.7	11.5	-	50.5	7.36	-	-	-	-						
14	Mixture 1 - PC-based	0.28	-	-	1069	-	0.9	0	0	0	0	0	0	0	0.0053	0	0	Polycarboxylate-based	43	9	-	38	5.9	-	-	-	-	-	-	10	200	40	[49]
	Mixture 1 - LP-based	0.56	-	-	-	-	1.28	0	0	1	0	0	0	0	0.008	0	0	Polycarboxylate-based	43	9	-	37	7.9	-	-	-	-	-	-				
	Mixture 1 - Combined	0.35	-	15	712.9	-	1	0	0	0.33	0	0	0	0	0.008	0	0	Polycarboxylate-based	43	9	-	39	7.8	-	-	-	-	-	-				
15	Ultra-high performance mortar with PP Le et al., (2012)	0.26	-	-	850	100	1.5	0.2	0.1	0	0	0	0	0	0.01	0.005	0	Polycarboxylate-based	107	11	-	93	13	-	-	-	-	-	-	-	-	-	[27]
	3D printable geopolymer mortar Panda. et al., (2017)	-	-	-	-	-	-	-	-	0	0	0	0	0	-	-	0	-	35	7.55	-	34	4.67	-	-	-	-	-	-	-	-	-	
	Geopolymer+ 0.25% PP Panda et al., (2017)	0.16	-	-	-	-	1.52	0.23	0.03	0	0	0	0	0.05	0	0.02	0	-	35	-	-	23.9	-	-	-	-	-	-	-	-	-	-	
	Geopolymer+ 0.5% PP Panda et al., (2017)	0.16	-	-	-	-	1.52	0.23	0.03	0	0	0	0	0.05	0	0.02	0	-	35	-	-	21.5	-	-	-	-	-	-	-	-	-	-	
	Geopolymer+ 0.75% PP Panda et al., (2017)	0.16	-	-	-	-	1.52	0.23	0.03	0	0	0	0	0.05	0	0.02	0	-	35	-	-	22.4	-	-	-	-	-	-	-	-	-	-	
	Geopolymer+ 1.0% PP Panda et al., (2017)	0.16	-	-	-	-	1.52	0.23	0.03	0	0	0	0	0.05	0	0.02	0	-	35	-	-	20.9	-	-	-	-	-	-	-	-	-	-	
	Cement based mortar Jianchao et al., (2017)	0.35	-	45	-	-	1	0	0	0	0	0	0	-	-	-	0	-	58.3	-	-	55.6	-	-	-	-	-	-	-	-	-	-	
	Geopolymer Paul et al., (2018)	-	-	-	-	30	1.53	-	-	-	-	-	-	-	-	-	-	-	35.9	8.59	-	36	9.66	-	-	-	-	-	-	-	-	-	
	3D printable CEM mortar Paul et al., (2018)	0.4	-	-	290	60	1.7	0.96	0.5	0	0	0	0	0	0.024	0	0	Sodium lignosulfonate (plasticizer)	51	-	-	47.9	-	-	-	-	-	-	-	-	-	-	
	3D printable CEM mortar + GF Paul et al., (2018)	0.4	-	-	289	60	1.7	0.96	0.5	0	0	0	0	0	0.031	0	0	Sodium lignosulfonate (plasticizer)	49.2	-	-	52.1	-	-	-	-	-	-	-	-	-	-	
	Printable cement mortar+fly ash Nerella et al., (2019)	0.26	-	-	430	90	1.8	0.4	0.21	0	0	0	0	0	0.023	0	0	Polycarboxylate-based	73.4	5.1	-	80.6	5.8	-	-	-	-	-	-	-	-	-	
	3D printable CEM mortar with 1 mm aggregate Wolfs et al., (2019)	0.495	-	90	-	60	-	-	-	0	0	0	0	0	-	-	-	-	42.3	4.28	-	28.51	4.7	-	-	-	-	-	-	-	-	-	
	3D Geopolymer mortar + 0.5 AN + 0.5 GD Bong et al., (2021)	0.36	-	291	727	65	1.5	0.5	0	0	0	0	0	0.5	0	0.15	0	Anhydrous sodium metasilicate powder, GD Grade sodium silicate powder and Sucrose powder	61.2	-	-	44.7	-	-	-	-	-	-	-	-	-	-	
	3D printing CEM mortar + PP Ma et al. (2018)	0.27	-	-	700	-	1.2	0.29	0.14	0	0	0	0	0	0.018	0	-	-	-	4.6	-	-	5.03	-	-	-	-	-	-	-	-	-	
	3D printable CEM mortar + BF Ma et al., (2019)	0.38	-	-	-	-	1.2	0.29	0.14	0	0	0	0	0	0.018	0	-	-	-	4.17	-	-	6.42	-	-	-	-	-	-	-	-	-	
16	[illegible] cement, the selected ground river-sand is with a fineness modulus of 2.3 and a maximum particle size of 1 mm, and the self-synthesized polycarboxylate superplasticizer [illegible]	0.32	-	-	840	-	0.67	0	0	0	0	0.08	0.03	0	0.03	0	0	Polycarboxylate	51.2	18.41	-	23.5	10.44	-	-	-	-	-	-	-	37.9	Area 1256.64 mm^2	[50]

17	Component kg/m3 - CEM I 52,5 R 550 - Fly Ash 250 - Water 280 - Aggregate 1172	0.35	172	135	550	90	1.47	0.45	0	0	0	0	0	0	-	-	-	-	65.8	8	4.6	63.17	7.8	1.8	-	-	22 °C	-	-	-	25	45	[51]
		0.35	172	135	550	90	1.47	0.45	0	0	0	0	0	0	-	-	-	-	-	6	-	-	3.9	-	-	-		-	-	-			
18	614 g Ordinary Portland Cement (PA-L 42.5), 68 g silica fume, 850 g rounded quartz sand having a particle size distribution ranging between 0 and 2 mm, water to cement ratio (W/C) equal to 0.4, and...	0.4	-	-	614	-	1.25	0	0.111	0	0	0	0	0	0.003	0	0	Polycarboxylate-based	55.2	1.51	-	46	0.97	-	-	-	22 °C	-	40	10	-	-	[52]
19	Water to binder ratio (w/b) of 0.33, sand to binder ratio (s/b) of 0.71, and aggregate to binder ratio (a/b) of 1.72. Polyethylene fibers were added by a volume fraction of binder equal to 1.4%	0.33	-	30	-	30	0.71	0	-	0	0	0	0	0	-	0	0.014	Polycarboxylate-based (MasterGlenium ACE 456) Not explicitly stated (only mentioned as "used" without % or ratio)	44.92	5.6	4.27 (T 30S)	22.00 (S1)	5.94 (S1)	3.15 (S1)	-	-	24 ± 2o C (Scenario S1) 40–45o C (Scenario S2)	-	40	20	-	-	[53]
		0.33	-	30	-	30	0.71	0	-	0	0	0	0	0	-	-	-	-	51.54	5.42	6.89	22	6.93	4.6	-	-		-			-	-	
		0.33	-	30	-	30	0.71	0	-	0	0	0	0	0	-	-	-	-	44.68	8.04	5.98	24	6.15	4.88	-	-		-			-	-	
		0.33	-	30	-	30	0.71	0	-	0	0	0	0	0	-	-	-	-	44.92	5.6	1.07 (T 2.0 hrs)	27.61	5.35	3.37	-	-		-			-	-	
		0.33	-	30	-	30	0.71	0	-	0	0	0	0	0	-	-	-	-	51.54	5.42	0.58	29.34	6.5	3.22	-	-		-			-	-	
		0.33	-	30	-	30	0.71	0	-	0	0	0	0	0	-	-	-	-	44.68	8.04	0.96	22	6.24	3.71	-	-		-			-	-	
		0.33	-	30	-	30	0.71	0	-	0	0	0	0	0	-	-	-	-	44.92	5.6	0 (T 4.0 hrs)	20.41 (S2)	5.68	2.36	-	-		-			-	-	
		0.33	-	30	-	30	0.71	0	-	0	0	0	0	0	-	-	-	-	51.54	5.42	0	14.8	6.75	2.63	-	-		-			-	-	
		0.33	-	30	-	30	0.71	0	-	0	0	0	0	0	-	-	-	-	44.68	8.04	0	22.57	7.96 (S2)	2.82	-	-		-			-	-	
		0.33	-	30	-	30	0.71	0	-	0	0	0	0	0	-	-	-	-	44.92	5.6	4.27 (T 30S)	19.83	5.48	1.60 (S2)	-	-		-			-	-	
		0.33	-	30	-	30	0.71	0	-	0	0	0	0	0	-	-	-	-	51.54	5.42	6.89	23.25	8.85	1.15	-	-		-			-	-	
		0.33	-	30	-	30	0.71	0	-	0	0	0	0	0	-	-	-	-	44.68	8.04	5.98	23.05	6.6	1.66	-	-		-			-	-	
		0.33	-	30	-	30	0.71	0	-	0	0	0	0	0	-	-	-	-	-	5.6	1.07 (T 2.0 hrs)	-	7.58	1.63	-	-		-			-	-	
		0.33	-	30	-	30	0.71	0	-	0	0	0	0	0	-	-	-	-	-	5.42	0.58	-	7.6	1.88	-	-		-			-	-	
		0.33	-	30	-	30	0.71	0	-	0	0	0	0	0	-	-	-	-	-	8.04	0.96	-	8.46	0.63	-	-		-			-	-	
		0.33	-	30	-	30	0.71	0	-	0	0	0	0	0	-	-	-	-	-	5.6	0 (T 4.0 hrs)	-	7.45	1.35	-	-		-			-	-	
		0.33	-	30	-	30	0.71	0	-	0	0	0	0	0	-	-	-	-	-	-	0	-	-	1.59	-	-		-			-	-	
		0.33	-	30	-	30	0.71	0	-	0	0	0	0	0	-	-	-	-	-	-	0	-	-	1.13	-	-		-			-	-	
20	Calcareous crushed sand with a grain size in the range of 0 to 2 mm, Ordinary Portland Cement (OPC) at a water to cement ratio of 0.4, 8% substitution of microsilica, and 15% substitution of limestone powder. A liquid superplasticizer is dosed at 0.7% by weight of the binder	0.4	-	-	-	480		0	0.08	0.15	0	0	0	0	0.0091	0.1	0	Polycarboxylate	56.5	9.7	-	64.7 (a)	6.3 (a)	-	-	-	20o C	Prefabrication offers access to well calibrated and accurate machines, it provides controlled curing environment for concrete.	-	4	180	-	[26]
		0.4	-	-	-	480		0	0.08	0.15	0	0	0	0	0.0091	0.1	0	Polycarboxylate	56.5	9.7	-	66.2 (b)	6.0 (b)	-	-	-			-			-	
		0.4	-	-	-	480		0	0.08	0.15	0	0	0	0	0.0091	0.1	0	Polycarboxylate	56.5	9.7	-	75.4 (c)	6.2 (c)	-	-	-			-			-	
		0.4	-	-	-	480		0	0.08	0.15	0	0	0	0	0.0091	0.1	0	Polycarboxylate	56.5	9.7	-	67.2 (d)	6.9 (d)	-	-	-			-			-	
21	Mix 1	0.4	145	-	614.5	-	1.25	0	0.11	0	0	0	0	0	0.0026	0	0	Superplasticizer (PCE-based)	77.4	-	-	49.1	-	-	-	-	-	-	-	-	-	10	[54]
	Mix 2	0.4	160	-	614.5	-	1.25	0	0.11	0	0	0	0	0	0.0036	0	0	Superplasticizer (PCE-based)	72.5	-	-	46.6	-	-	-	-	-	-	-	-	-		
	Mix 3	0.4	180	-	614.5	-	1.25	0	0.11	0	0	0	0	0	0.004	0	0	Superplasticizer (PCE-based)	71.9	-	-	46.5	-	-	-	-	-	-	-	-	-		
	Mix 4	0.45	200	-	614.5	-	1.25	0	0.11	0	0	0	0	0	0.004	0	0	Superplasticizer (PCE-based)	56.5	-	-	46.7	-	-	-	-	-	-	-	-	-		
22	Ordinary Portland cement was used for the mixture. Additionally, two types of cementitious materials, namely, silica fume and class C fly ash. SF with a SiO2 content of 91.3% and Class C FA used	0.29	148	-	576	-	1.4	0.299	0.137	0	0	0	0	0	0	0.0144	0	High-Performance Water-Reducing Agent	72.8	11.9	-	23.5	6.1	-	-	-	-	A water tank until a curing age of 28 days	40	10	100 & 80	40	[55]
23	CEM I 42.5 R grey cement - cement: water: fine sand (0–1.5 mm): chemical additive = 1: 0.30: 1.5: 0.02. 800 kg/m3 cement. 1.5% and 2% of the cement weight water-reducing admixture SA	0.3	170	-	800	60	1.5	0	0	0	0	0	0	0	1.5% to 2.0%	0.003	0	-	59.8	9.4	-	57.9	9.1	-	-	-	-	-	40, 50	-	-	32, 40 and 50	[56]
		0.3	170	-	800	60	1.5	0	0	0	0	0	0	0	1.5% to 2.0%	0.003	0	-	59.8	-	-	56.7	7.9	-	-	-	-	-		-	-		
		0.3	170	-	800	60	1.5	0	0	0	0	0	0	0	1.5% to 2.0%	0.003	0	-	59.8	-	-	57.1	8.2	-	-	-	-	-		-	-		
	CEM I 52.5 R white cement - cement: water: fine sand (0–1.5 mm): chemical additive = 1: 0.30: 1.5: 0.02. 800 kg/m3 cement. 1.5% and 2% of the cement weight water-reducing admixture SA	0.3	190	-	800	60	1.5	0	0	0	0	0	0	0	1.5% to 2.0%	0.003	0	-	64	11.8	-	62.8	11.4	-	-	-	-	-		-	-		
		0.3	190	-	800	60	1.5	0	0	0	0	0	0	0	1.5% to 2.0%	0.003	0	-	64	-	-	61.5	10.1	-	-	-	-	-		-	-		
		0.3	190	-	800	60	1.5	0	0	0	0	0	0	0	1.5% to 2.0%	0.003	0	-	64	-	-	61.9	10.5	-	-	-	-	-		-	-		
		0.3	190	-	800	60	1.5	0	0	0	0	0	0	0	1.5% to 2.0%	0.003	0	-	59.8	-	-	57.9	-	-	-	-	-	-		-	-		
		0.3	190	-	800	60	1.5	0	0	0	0	0	0	0	1.5% to 2.0%	0.003	0	-	59.8	-	-	56.7	-	-	-	-	-	-		-	-		
		0.3	190	-	800	60	1.5	0	0	0	0	0	0	0	1.5% to 2.0%	0.003	0	-	59.8	-	-	57.1	-	-	-	-	-	-		-	-		
24	SF Mix	0.32	-	-	574	-	1.5	0.2	0.1	0	0	0	0	0	0.0024	0	0.0031	Superplasticizer (PCE-based)	74	-	-	62	6	6.1	-	-	25 - 30	-	-	-	44	30 x 30	[57]
	NC Mix	0.32	-	-	663	-	1.5	0.2	0.1	0	0	0	0	0	0.0015	0	0.0027	Superplasticizer (PCE-based)	72	-	-	62	6.5	5.9	-	-		-	-	-			
	VM Mix	0.32	-	-	663	-	1.5	0.2	0.1	0	0	0	0	0	0.0021	0.0012	0.0027	Superplasticizer (PCE-based)	68	-	-	60	6.25	5.5	-	-		-	-	-			
25	-	0.26	-	-	550	-	1.2	0.29	0.14	0	0	0	0	0	0.018	0	0	Polycarboxylate-based	39.5	-	-	39.6	5.03	6.42	-	-	20	-	-	5	75	12	[58]
	-	0.26	-	-	550	-	1.2	0.29	0.14	0	0	0	0	0	0.018	0	0	Polycarboxylate-based	39.5	-	-	29.8	5.03	-	-	-		-	-				
	-	0.26	-	-	550	-	1.2	0.29	0.14	0	0	0	0	0	0.018	0	0	Polycarboxylate-based	74	-	-	-	10.7	-	-	-		-	-				
	-	0.26	-	-	550	-	1.2	0.29	0.14	0	0	0	0	0	0.018	0	0	Polycarboxylate-based	72	-	-	-	11.5	-	-	-		-	-				
	-	0.26	-	-	550	-	1.2	0.29	0.14	0	0	0	0	0	0.018	0	0	Polycarboxylate-based	68	-	-	-	10.6	-	-	-		-	-				
	-	0.26	-	-	550	-	1.2	0.29	0.14	0	0	0	0	0	0.018	0	0	Polycarboxylate-based	52.5	-	-	-	6	-	-	-		-	-				
26	Portland cement (CEM I 52.5 R), siliceous aggregate with a maximum particle size of 1 mm, limestone filler, additives, rheology modifiers and a small amount of polypropylene (PP) fibres. The water to fines (cement+fillers) ratio equals 0.495.	0.495	-	60	-	60	-	0	0	0	0	0	0	0	-	-	-	-	42.3	4.28	-	28.51	4.29	-	-	-	24	All the samples were stored in water to maintain an equal curing history	50	9.5	-	-	[59]
		0.495	-	60	-	60	-	0	0	0	0	0	0	0	-	-	-	-	42.3	4.28	-	29.19	4.7	-	-	-					-	-	
		0.495	-	60	-	60	-	0	0	0	0	0	0	0	-	-	-	-	-	4.28	-	-	3.68	-	-	-					-	-	
		0.495	-	60	-	60	-	0	0	0	0	0	0	0	-	-	-	-	-	4.28	-	-	3.51	-	-	-					-	-	
		0.495	-	60	-	60	-	0	0	0	0	0	0	0	-	-	-	-	-	4.28	-	-	3.48	-	-	-					-	-	
		0.495	-	60	-	60	-	0	0	0	0	0	0	0	-	-	-	-	-	4.28	-	-	3.34	-	-	-					-	-	
		0.495	-	60	-	60	-	0	0	0	0	0	0	0	-	-	-	-	-	4.28	-	-	3.1	-	-	-					-	-	
		0.495	-	60	-	60	-	0	0	0	0	0	0	0	-	-	-	-	-	4.28	-	-	3.92	-	-	-					-	-	
		0.495	-	60	-	60	-	0	0	0	0	0	0	0	-	-	-	-	-	4.28	-	-	3.98	-	-	-					-	-	
		0.495	-	60	-	60	-	0	0	0	0	0	0	0	-	-	-	-	-	4.28	-	-	2.65	-	-	-					-	-	
		0.495	-	60	-	60	-	0	0	0	0	0	0	0	-	-	-	-	-	4.28	-	-	2.35	-	-	-					-	-	
		0.495	-	60	-	60	-	0	0	0	0	0	0	0	-	-	-	-	-	4.28	-	-	1.72	-	-	-					-	-	
		0.495	-	60	-	60	-	0	0	0	0	0	0	0	-	-	-	-	-	4.28	-	-	2.28	-	-	-					-	-	
27	60% calcium sufflaminate (CSA) and 40% type I ordinary Portland cement (OPC) by mass. Natural Sand River of 4.75 mm maximum size and w/c = 0.50	0.5	-	45	768	16	1.5	0	0	0	1.5	0	0	0	-	-	0	-	26.12	-	-	19.34	-	-	-	-	Room temperature 20	-	-	-	-	-	[60]
		0.5	-	45	768	16	1.5	0	0	0	1.5	0	0	0	-	-	0	-	27.03	-	-	20.84	-	-	-	-		-	-	-	-	-	
		0.5	-	45	768	16	1.5	0	0	0	1.5	0	0	0	-	-	0	-	25.41	-	-	21	-	-	-	-		-	-	-	-	-	
		0.5	-	45	768	16	1.5	0	0	0	1.5	0	0	0	-	-	0	-	26.12	-	-	22.59	-	-	-	-		-	-	-	-	-	
		0.5	-	45	768	16	1.5	0	0	0	1.5	0	0	0	-	-	0	-	27.03	-	-	23.17	-	-	-	-		-	-	-	-	-	
		0.5	-	45	768	16	1.5	0	0	0	1.5	0	0	0	-	-	0	-	25.41	-	-	24.12	-	-	-	-		-	-	-	-	-	
		0.5	-	45	768	16	1.5	0	0	0	1.5	0	0	0	-	-	0	-	26.12	-	-	22.15	-	-	-	-		-	-	-	-	-	
		0.5	-	45	768	16	1.5	0	0	0	1.5	0	0	0	-	-	0	-	27.03	-	-	20.86	-	-	-	-		-	-	-	-	-	
		0.5	-	45	768	16	1.5	0	0	0	1.5	0	0	0	-	-	0	-	25.41	-	-	22.52	-	-	-	-		-	-	-	-	-	
		0.5	-	45	768	16	1.5	0	0	0	1.5	0	0	0	-	-	0	-	26.12	-	-	21.36	-	-	-	-		-	-	-	-	-	
		0.5	-	45	768	16	1.5	0	0	0	1.5	0	0	0	-	-	0	-	27.03	-	-	19.92	-	-	-	-		-	-	-	-	-	
		0.5	-	45	768	16	1.5	0	0	0	1.5	0	0	0	-	-	0	-	25.41	-	-	20.53	-	-	-	-		-	-	-	-	-	
28	-	0.26	-	-	477	100	1.5	0.28	0.14	0	0	0	0	0	0.01	0.005	0.00186	Polycarboxylate-based	107	11	-	75	6	-	-	-	-	The components were then covered with damp hessian and plastic sheeting for a day before being moved to a laboratory storage area where the same curing regime was maintained up to 28 days.	-	-	-	9	[61]
29		0.26	-	-	477	100	1.5	0.28	0.14	0	0	0	0	0	0.01	0.005	0.00186	Polycarboxylate-based	107	11	-	102	17	-	-	-	-		-	-	-		

Abbreviations: W/B = Water-to-Binder ratio; IST = Initial Setting Time; FA = Fly Ash; SF = Silica Fume; LP = Limestone Powder; CSA = Calcium Sulfoaluminate; NA = Nano Attapulgite; HPMC = Hydroxypropyl Methylcellulose; Slag = Ground Granulated Blast-Furnace Slag (GGBS); SP = Superplasticizer; Acc/Ret = Accelerator and Retarder; PE = Polyethylene; Comp. = Compressive Strength; Tens. = Tensile (Flexural) Strength; 3DCP = 3D Concrete Printing; E = Young's Modulus; Temp = Temperature; Layer W/H = Layer Width/Height; Nozzle Diam. = Nozzle Diameter; Ref = Reference number (see reference list below).
